# Development of a climate-sensitive disease forecasting tool in the Horn of Africa: A multi-stakeholder exploration of priorities in Ethiopia, Kenya, Somalia, and Uganda

**DOI:** 10.1371/journal.pgph.0005634

**Published:** 2026-11-10

**Authors:** Tessa Rose Cornell, Clovice Kankya, Mulugeta Tamire, Jane N. Nyambura, Yusuf Abdi Hared, John Baptist Galiwango, Davis Lois Nagadya, Rose Ayebale, Siobhan M. Mor, Andrew P. Morse, Cyril Caminade, Matthew Baylis, Louise A. Kelly-Hope, Eric M. Fèvre

**Affiliations:** 1 Institute of Infection, Veterinary and Ecological Sciences (IVES), University of Liverpool, Liverpool, United Kingdom; 2 Department of Biosecurity, Ecosystems and Veterinary Public Health (BEP), College of Veterinary Medicine, Animal Resources and Biosecurity (COVAB), Makerere University, Kampala, Uganda; 3 Department of Preventive Medicine, School of Public Health, Addis Ababa University, Addis Ababa, Ethiopia; 4 Department of Community Health, School of Public Health, Amref International University, Nairobi, Kenya; 5 School of Health and Welfare, Dalarna University, Falun, Sweden; 6 Amoud University College of Health Sciences, Borama, Somaliland, Somalia; 7 International Livestock Research Institute (ILRI), Nairobi, Kenya; 8 Department of Geography and Planning, School of Environmental Sciences, University of Liverpool, Liverpool, United Kingdom; 9 Earth System Physics Department, Abdus Salam International Centre for Theoretical Physics (ICTP), Trieste, Italy; New York University Grossman School of Medicine, UNITED STATES OF AMERICA

## Abstract

Extreme climate events impose significant impacts across the Horn of Africa. One way to monitor these is by using novel digital tools to forecast extreme events and climate-sensitive disease outbreaks. However, limited information exists on stakeholder use of such tools. This qualitative study identified key multidisciplinary stakeholders in Ethiopia, Kenya, Somalia, and Uganda, and conducted a needs assessment and gap analysis. Focal persons of multi-lateral agencies, government institutions and ministries, non-governmental organisations, and academic institutions, at regional, national, and sub-national levels, were engaged in Key Informant Interviews (KIIs; *N* = 63) and Focus Group Discussions (FGDs; *N* = 7, total participants = 57). Malaria (54.0%), Rift Valley Fever (RVF; 44.4%), cholera (39.7%), dengue fever (27.0%), and anthrax (14.3%) were the most frequently reported climate-sensitive disease priorities amongst KII participants. Country-level variations existed in the use of climate or environmental data to support disease decision-making (Kenya: 95.2% of KII participants, Uganda: 75.0%, Somalia: 64.3%, Ethiopia: 58.3%), electronic reporting systems (Kenya: 42.9%, Ethiopia: 41.7%, Somalia: 14.3%, Uganda: 12.5%), and modelled outputs (Kenya: 57.1%, Uganda: 25.0%, Somalia: 7.1%). FGD participants described barriers to sustainable digitalised systems including cost-effectiveness, lack of technological expertise or support, and poor Internet access, which may be addressed by engaging key national stakeholders, training provision, and financial resources. Overall, digital tool preferences for analysing, displaying or reporting data included provision of data summaries and visual aids, a dashboard format, and functions to download data for further analysis. This study provides critical insights into the needs of disease control stakeholders who would benefit from, and are the likely primary users of, digital tools to tackle the increasing threat of climate-sensitive diseases. It underscores the importance of applying context-specific approaches to supporting local capacity, which will inform the development of a sustainable Climate Sensitive Disease Forecasting Tool (CLIMSEDIS).

## 1 Introduction

The Horn of Africa is particularly vulnerable to the impacts of extreme climate events with climate change predictions including warming oceans and increasing temperatures and rainfall variations [1]. In 2019, cyclones, record rainfall, and floods across East Africa (including countries within the Horn of Africa) resulted in widespread food insecurity, population displacement, and increased incidence of vector-borne (e.g., malaria, Rift Valley fever (RVF), and dengue fever) and waterborne (e.g., cholera and typhoid fever) diseases [2,3]. The Horn of Africa also experiences severe recurrent droughts, resulting in food and water shortages which lead to human and animal starvation [4].

An understanding of drivers of climate-sensitive disease distributions and outbreaks can help to inform national strategies for emergency preparedness and response [5,6]. A range of open-access web-based sources provide historic and current environmental data or near-real time climate forecasts, using a combination of satellite-based data and models [7–13]. Exploiting these resources and understanding the spatial and temporal overlap with priority climate-sensitive diseases are crucial to support both climate mitigation and adaptation strategies, and disease risk assessments, models, and preparatory measures [14].

Improved global internet accessibility provides opportunities to develop digital tools that help key stakeholders predict, prepare, and respond more effectively to disease outbreaks. Digital tools have been shown to improve the distribution of human and financial resources, and to inform the design of timely public or livestock health interventions [15–18]. Key stakeholders include policymakers and disease control programme teams at national, sub-national, and regional levels (e.g., the World Health Organization (WHO) regional offices, government ministries dedicated to human, animal, and environmental or climate health, local government responsible for deploying resources to their populations, and non-governmental organisations (NGOs)).

Overall, there is a lack of digital tools to support decision making for climate-sensitive infectious diseases, which has led to specific research opportunities to target this gap [19]. For any tool to be effective, it is critical to understand stakeholders’ roles and responsibilities, and their potential usage of any tool, perceptions of its usefulness, and intention and willingness to adopt it (where adoption implies the potential to make different decisions because of the use of the tool). In the Horn of Africa, there is limited information on stakeholder use or willingness to use climate data services and digital tools to forecast extreme events and disease outbreaks.

To inform the development of the innovative Climate Sensitive Disease Forecasting Tool (CLIMSEDIS) for the Horn of Africa [20], the primary aims of this study were to: (i) identify key multidisciplinary stakeholders, with a focus on Ethiopia, Kenya, Somalia, and Uganda; and (ii) conduct a needs assessment and gap analysis.

## 2 Methods

### 2.1 Ethics statement

Ethical approval for the research was obtained from the University of Liverpool Research Ethics Committee (14258), and from national ethical approval bodies, as follows: the Ethiopian Public Health Association Institutional Review Board (EPHA/OG/504/24), Ethiopia; ILRI Institutional Research Ethics Committee (ILRI-IREC2023-72/1), Kenya; College of Health Sciences Research and Ethics committee (MAKSHSREC-2024-757) and the Uganda National Council for Science and Technology (HS4929ES), Uganda; and Amoud University Research Ethical Committee (0886-AU-REC-2024), Somalia.

Participants’ personal identifiers were kept strictly confidential with access restricted to the country-level collaborators (CK, MT, JNN, YAH, JG, DLN, RA) and University of Liverpool researchers (TRC, EMF, SMM, LAKH) who collected and analysed the data. Participants were assigned unique identifiers to maintain confidentiality. Anonymised transcripts, completed questionnaire survey forms, and Microsoft Excel data files, were stored securely with access restricted to the research team.

### 2.2 Study areas

The four countries selected within the Horn of Africa were Ethiopia, Kenya, Somalia (with a focus on Somaliland), and Uganda, where long-standing research collaborations and networks with University of Liverpool researchers had been previously established. The selection of these countries also provided opportunities to compare the common and unique needs and gaps across landscapes with differing infectious disease co-endemicity and diverse geoclimatic characteristics as shown in Fig 1 [21].

**Fig 1:**
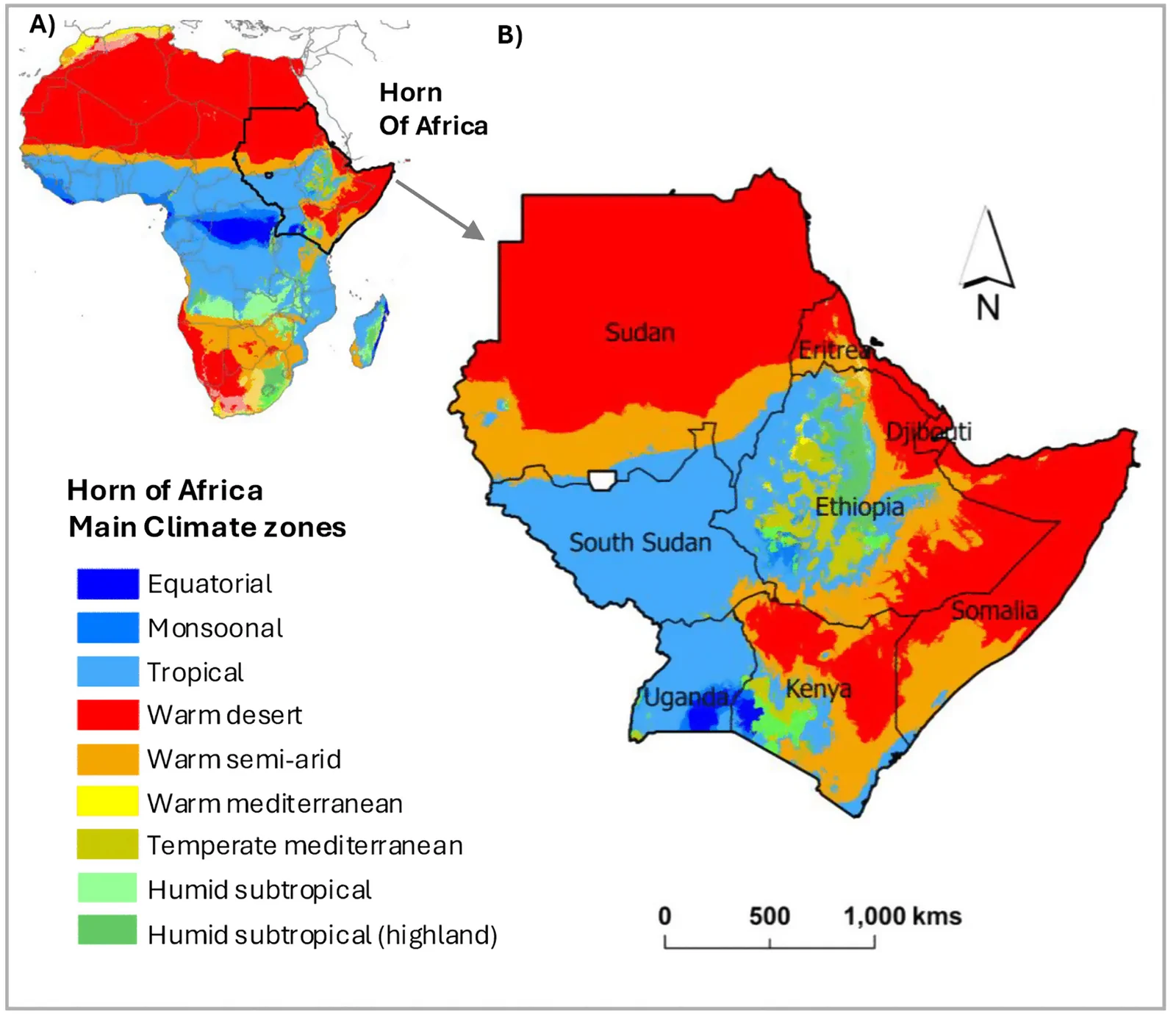
Horn of Africa region and climate zones: A) Climate zones across Africa; B) Horn of Africa study region. Geo-referenced data available from https://www.gloh2o.org/koppen/ (access date 15/10/2025) under the CC BY 4.0 license, with attribution to Beck *et al.* (2018) [21]. The legend was adapted to highlight the main climate zones for the Horn of Africa region. Administrative boundary data and base layer of the map are available from https://www.geoboundaries.org/ (access date 16/04/2026) under the CC BY 4.0 license, with attribution to Runfola *et al.* (2020) [22].

### 2.3 Study design and stakeholder selection

To conduct a needs assessment and gap analysis amongst key stakeholder groups, a qualitative study design was applied, which comprised semi-structured Key Informant Interviews (KIIs) and Focus Group Discussions (FGDs). KIIs and FGDs were coordinated by researchers and consultants with training or experience in qualitative data collection in the selected countries, and with research interests in climate-sensitive infectious diseases (CK, MT, JNN, YAH, JG). The Consolidated Criteria for Reporting Qualitative Studies (COREQ) checklist was used to guide reported outcomes (**S1 Checklist**).

In each of the selected countries, purposive and snowball sampling approaches were applied to identify focal persons of multi-lateral agencies, government institutions and ministries, non-governmental organisations (NGOs), and academic institutions, at regional, national, and sub-national levels. The inclusion criteria for key informants and organisations are detailed in **Table 1**.

**Table 1:** Inclusion criteria for stakeholder and organisation selection.

Organisations	Individuals
Focus on public health, climate-related issues, or climate change initiatives.	Decision-making authority and expertise in implementing disease control strategies, policies, and community engagement.
Access to relevant datasets (including epidemiological, climate, or environmental data).	Responsible for disease surveillance and control activities.
	Good understanding of the organisational structures of the identified organisations to identify key decision-makers.
	Subject matter experts.

An introductory email or letter was sent to identified key informants. Voluntary informed consent was sought from each participant, including provision of study information and explanation of data management and anonymisation procedures. Participants provided signed or recorded verbal consent, in accordance with national ethical requirements, and in-person or online KIIs and FGDs were scheduled.

### 2.4 Data collection

#### 2.4.1 Key informant interviews.

KIIs aimed to capture individual stakeholder perspectives and insights. Interviews were held in-person at the participants’ work address or via online platforms Microsoft Teams (Microsoft Corporation, 2025) or Google Meet™ video conferencing system in 2024 and 2025 (Ethiopia: 03/10/2024-16/01/2025; Kenya: 13/06/2024–30/10/2024; Somalia: 20/11/2024-06/01/2025; Uganda: 18/07/2024–30/09/2024). The KIIs were recorded using a handheld device or the online platform recording function. Interviews were held in English or the preferred local language (Amharic in Ethiopia and Somali in Somalia) and lasted approximately 0.5-1 hour. Only the researchers and participants were present during KIIs.

A semi-structured KII question guide (**S1 File**), in addition to follow-up and probe questions, addressed the question topics summarised in **Table 2**. Original audio files were stored securely on laptop devices, with access restricted to research team members in-country (CK, MT, JNN, YAH, JG, DLN, RA).

**Table 2:** Key Informant Interview and Focus Group Discussion question topics and sub-topics.

Question theme	Sub-topics
Stakeholders	
**Stakeholder Characteristics**	• Country •Organisation or programme affiliation(s) • Populations served
**Current Situation**	
**Infectious Diseases of Primary Concern**	• Priority diseases • Disease preparedness challenges and solutions
**Disease Risk in Relation to ENSO and Seasonality**	• ENSO awareness • Seasonality consideration
**Decision-Making Processes**	• Institutional level of decision-making and communicating decisions on future disease risk • Relationship between national and sub-national decision-making
**Existing Data to Support Decision-Making**	• Data types and sources to inform future disease risk • Climate or environmental data uses and sources
**Interventions and Strategies Informed by Existing Data**	• Examples and outcomes of decisions on future disease risk
**Existing Digital Tools to Support Decision-Making**	• Digital tool interaction and device use • Reporting systems in outbreak response • Digital tool use • Use of modelled output
**Future Tools**	
**Digital Tool Format and Presentation Preferences**	• Digital tool format preferences • Device type preferences • Disease data types • Geographical resolution • Level of digital tool interaction
**Optimal Lead-Time**	• Optimal lead-time to receive information
**Digital Tool Sustainability**	• Barriers and solutions

Acronym: ENSO = El Niño-Southern Oscillation.

#### 2.4.2 Focus Group Discussions (FGDs).

FGDs aimed to capture shared discussions and information exchange amongst participants in organisations and institutions responsible for disease surveillance and control. FGD participants provided national-, sub-national, or institutional-level perspectives.FGDs were conducted in-person at the participants’ work address or via online platforms Microsoft Teams (Microsoft Corporation, 2025) or Google Meet™ video conferencing system in 2024 and 2025 (Ethiopia: 03/10/2024-16/01/2025; Somalia: 26/11/2024-22/04/2025; Uganda: 08/01/2025-15/05/2025). They were recorded using a handheld device or the online platform recording function. Interviews were held in English or the preferred local language and lasted approximately 1–2 hours. A FGD question guide, in addition to follow-up and probe questions, addressed the question topics described in **Table 2**. Audio-recordings were complemented by supplementary hand-written notes to capture key points and non-verbal cues. Only the researchers and participants were present during FGDs.

### 2.5 Data management and analysis

KIIs and FGDs held in local languages were translated into English and all results were transcribed *verbatim* manually or using transcription software (© 2025 Otter.ai Inc), followed by an additional manual review to check transcript accuracy. Transcripts were analysed by manual thematic analysis, using pre-determined question topics and sub-topics. Relevant transcript sections were copied into a template Microsoft Excel form. Data from KIIs and FGDs were summarised by key topics and sub-topics. Preliminary data management and thematic analysis were performed by country-level collaborators (CK, MT, JNN, YAH, JG, DLN, RA). Direct quotes were extracted from transcripts to enrich quantitative outcomes, including key inter-country variations. Subsequent collation of datasets, data coding and analysis, and generation of graphics were performed using Microsoft Excel and IBM SPSS Statistics 29.0 (TRC, LAKH). Identified themes relating to pre-determined question topics were categorised and summarised using descriptive statistics.

## 3. Results

### 3.1 Stakeholder characteristics

#### 3.1.1 Key informant interview (KII) participants.

A total of 63 KIIs were conducted with stakeholders in Ethiopia (*n* = 12, 19.0%), Kenya (*n* = 21, 33.3%), Somalia (*n* = 14, 22.2%), and Uganda (*n* = 16, 25.4%). Stakeholder affiliations included government ministries and institutions (*n* = 29/63, 46.0%), NGOs or non-profit organisations (*n* = 11/63, 17.5%), multi-lateral agencies (*n* = 9/63, 14.3%), academia (*n* = 7/63, 11.1%), and international research organisations (*n* = 4/63, 6.3%; Table A in S1 Appendix). Across all countries, the most frequently reported affiliation of stakeholders was government, encompassing ministries or institutions dedicated to health or public health (*n* = 14/29, 48.3%), veterinary science or agriculture (*n* = 8/29, 27.6%), environment, climate or water (*n* = 3/29, 10.3%), meteorology (*n* = 2/29, 6.9%), zoonotic diseases (*n* = 1), and disaster preparedness and food (*n* = 1).

Stakeholders reported serving both urban and rural populations (urban: *n* = 42/63, 66.7%; rural: *n* = 41/63, 65.1%). Other described populations included climate stakeholders, displaced or host communities, children under 5 years of age and pregnant women, and more broadly, underserved populations.

#### 3.1.2 Focus group discussion (FGD) participants.

FGDs were conducted in Ethiopia (*n* = 2, total participants = 13), Uganda (*n* = 2, total participants = 19), and Somalia (*n* = 3, total participants = 25; Table B in S1 Appendix). In Ethiopia, the majority of FGD participants were affiliated with academic institutions (*n* = 12/13, 92.3%). In Uganda, 94.7% of participants (*n* = 18/19) were affiliated with government ministries and institutions, including those dedicated to health or public health, veterinary or agriculture, environment, and One Health. In Somalia, participant affiliations comprised national government ministries and institutions including sub-national level participants (*n* = 13/25, 52.0%), NGOs and UN agencies (*n* = 9/25, 36.0%), and academic institutions (*n* = 3/25, 12.0%).

### 3.2 Infectious diseases of primary concern

#### 3.2.1 Priority diseases.

The most frequently reported climate-sensitive diseases of concern amongst KII participants were malaria (*n* = 34/63, 54.0%), RVF (*n* = 28/63, 44.4%), cholera (*n* = 25/63, 39.7%), dengue fever (*n* = 17/63, 27.0%), and anthrax (*n* = 9/63, 14.3%; **Table 3**). Country-level variations in the three most frequently reported priority diseases were documented as follows (in descending order of reporting frequency): Ethiopia – malaria, cholera, dengue fever; Kenya – RVF, malaria, dengue fever; Uganda – RVF, anthrax, malaria; and Somalia – cholera, malaria, dengue fever. Other climate-sensitive diseases mentioned encompassed priority human, animal, and plant diseases (**Table 3** footnotes).

**Table 3:** Climate-sensitive diseases reported by Key Informant Interview participants as organisational priority diseases of concern, categorised by (A) country and (B) stakeholder affiliation.

Stakeholder reports, *n* (%)	(A) Country										Total, *N*=63
	Ethiopia, *N*=12		Kenya, *N*=21			Somalia, *N*=14		Uganda, *N*=16			
Malaria	11 (91.7)		10 (47.6)			7 (53.8)		6 (37.5)			34 (54.0)
Rift Valley Fever	1 (8.3)		13 (61.9)			5 (35.7)		9 (56.3)			28 (44.4)
Cholera	9 (75.0)		3 (14.3)			8 (57.1)		5 (31.3)			25 (39.7)
Dengue fever	5 (41.7)		6 (28.6)			6 (42.9)		-			17 (27.0)
Anthrax	-		2 (9.5)			-		7 (43.8)			9 (14.3)
Crimean-Congo haemorrhagic fever	-		1 (4.8)			-		4 (25.0)			5 (7.9)
West Nile Virus	-		2 (9.5)			-		-			2 (3.2)
Chikungunya	2 (16.7)		2 (9.5)			1 (7.1)		1 (6.3)			6 (9.5)
Yellow fever	1 (8.3)		-			-		-			1 (1.6)
Typhoid fever	-		-			-		2 (12.5)			2 (3.2)
Respiratory infections	1 (8.3)		2 (9.5)			2 (14.3)		-			5 (7.9)
Other	3 (25.0) ^a^		7 (33.3) ^b^			8 (57.1) ^c^		6 (37.5) ^d^			24 (38.1)
**Stakeholder reports, *n* (%)**	**(B) Stakeholder Affiliations**										**Total, *N*=63**
	**Multi-lateral agency: UN, *N*=8**	**Multilateral agency: Non-UN, *N*=1**		**Government, *N*=29**	**Academia, *N*=7**		**International research organisation, *N*=4**	**NGO/ Non-profit organisation, *N*=11**	**Private sector, *N*=1**	**Multiple affiliations, *N*=2**	
Malaria	4 (50.0)	1 (100.0)		15 (51.7)	3 (42.9)		1 (25.0)	8 (72.7)	-	2 (100.0)	34 (54.0)
Rift Valley Fever	5 (62.5)	-		15 (51.7)	2 (28.6)		2 (50.0)	3 (27.2)	-	1 (50.0)	28 (44.4)
Cholera	4 (50.0)	1 (100.0)		12 (41.4)	1 (14.3)		-	7 (63.6)	-	-	25 (39.7)
Dengue fever	3 (37.5)	-		7 (24.1)	4 (57.1)		2 (50.0)	1 (9.1)	-	-	17 (27.0)
Anthrax	1 (12.5)	-		5 (17.2)	1 (14.3)		1 (25.0)	1 (9.1)	-	-	9 (14.3)
Chikungunya	-	-		4 (13.8)	2 (28.6)		-	-	-	-	6 (9.5)
CCHF	2 (25.0)	-		3 (10.3)	-		-	-	-	-	5 (7.9)
Respiratory infections	1 (12.5)	-		1 (3.4)	-		-	2 (18.2)	-	1 (50.0)	5 (7.9)
Typhoid fever	-	-		2 (6.9)	-		-	-	-	-	2 (3.2)
West Nile Virus	1 (12.5)	-		-	-		-	1 (9.1)	-	-	2 (3.2)
Yellow fever	-	-		1 (3.4)	-		-	-	-	-	1 (1.6)
Other	1 (12.5) ^e^	1 (100.0) ^f^		12 (41.4) ^g^	3 (42.9) ^h^		3 (75.0) ^i^	3 (27.2) ^j^	1 (100.0) ^k^	-	24 (38.1)

Acronyms: CCHF=Crimean Congo Haemorrhagic Fever; NGO=non-governmental organisation. The following frequencies may exceed frequencies stated for ‘Other’ diseases if individual participants have reported multiple diseases: ^a^ *Ethiopia*: Human trypanosomiasis (*n* = 1), Meningitis (*n* = 2); ^b^ *Kenya*: Bluetongue (*n* = 3), Nairobi sheep disease (*n* = 1), Tick borne diseases (*n* = 1), Contagious Bovine Pleuropneumonia (CBPP; *n* = 1), Schistosomiasis (*n* = 1), Q-fever (*n* = 1), Brucellosis (*n* = 1), Influenzas (*n* = 1); ^c^ *Somalia*: Desert locust infestation (*n* = 2), Leaf spot diseases (*n* = 1), Aflatoxin crop contamination (*n* = 1), Anaemia related to heat stress (*n* = 1), Parasitic infection (*n* = 3), Malnutrition (*n* = 5), Avian influenza (*n* = 1), Salmonellosis (in poultry, *n* = 1), Measles (*n* = 1), Tuberculosis (*n* = 1), Leishmaniasis (*n* = 1); ^d^ *Uganda*: Trypanosomiasis (*n* = 1), COVID-19 (*n* = 1), Helminthiasis (*n* = 1), Tick borne diseases (*n* = 1), Swine Fever (*n* = 1), Avian Influenzas (*n* = 1), Viral haemorrhagic fever (*n* = 1), Influenza (*n* = 1), African Swine Fever (*n* = 1), East Coast Fever (*n* = 1). ^e^ *Multi-lateral agency: UN:* Bluetongue (*n* = 1); ^f^ Meningitis (*n* = 1); ^g^ *Government:* Schistosomiasis (*n* = 1), Bluetongue (*n* = 2), Helminthiasis (*n* = 1), Influenza (*n* = 1), Meningitis (*n* = 1), Tick borne diseases (*n* = 2), Nairobi sheep disease (*n* = 1), CBPP (*n* = 1), Swine Fever (*n* = 1), Avian Influenzas (*n* = 1), Desert locust infestation (*n* = 1), Leaf spot diseases (*n* = 1), Aflatoxin crop contamination (*n* = 1), Anaemia related to heat stress (*n* = 1), Parasitic infection (*n* = 1), Malnutrition (*n* = 3); ^h^ *Academia*: (Human) trypanosomiasis (*n* = 2), COVID-19 (*n* = 1), Parasitic diseases (*n* = 1), Leishmaniasis (*n* = 1), Malnutrition (*n* = 1); ^i^ *International research organisation:* African Swine Fever (*n* = 1), East Coast Fever (*n* = 1), Influenzas (*n* = 1), Q-fever (*n* = 1), Brucellosis (*n* = 1); ^j^ *NGO/ Non-profit organisation:* Viral haemorrhagic fever (*n* = 1), Desert locust infestation (*n* = 1), Malnutrition (*n* = 1), Measles (*n* = 1), Tuberculosis (*n* = 1); ^k^ *Private sector*: Avian influenza (*n* = 1), Salmonellosis (in poultry, *n* = 1), Parasitic infections (*n* = 1).

Government ministries or institutions, which constituted the main stakeholder group, identified malaria (*n* = 15/29, 51.7%), RVF (*n* = 15/29, 51.7%), and cholera (*n* = 12/29, 41.4%) as the three primary diseases of concern (**Table 3**). These results were consistent with the priority diseases of concern reported by stakeholders affiliated with UN agencies and NGOs or non-profit organisations, which were RVF, malaria, and cholera for both stakeholder groups.

In Uganda, a consensus emerged amongst FGD participants that many infectious diseases in Uganda are climate sensitive. Human infectious diseases of concern included malaria, cholera, RVF, anthrax, Crimean-Congo Haemorrhagic Fever (CCHF), yellow fever, and dysentery. The range of diseases listed was broad and reflected variable priorities at national and sub-national levels and across diverse ecological zones. Participants in rural Kasese made a distinction between diseases of international concern and highly localised diseases.

*“…the changes in Climate in Kasese may not be the changes of Climate in Kisoro. For example the diarrheal diseases around here, which are not a problem in Kisoro because of the difference in rainfall amount received.”* [Uganda: FGD Kasese, P1]

Although FGD participants in Somalia highlighted vector-borne and zoonotic diseases as broad priorities, specific disease priorities varied by affiliation, with prioritisation of malaria and dengue fever by public health stakeholders, and RVF and brucellosis by stakeholders with an animal health focus.

#### 3.2.2 Disease preparedness challenges and solutions.

KII participants described a range of challenges related to climate-sensitive disease preparedness. The most frequently reported challenges related to the following themes: financial resources; data availability, access, quality, or sharing; information sharing, collaboration, or coordination between sectors or stakeholder groups; material resources (including diagnostics and vaccines); human resources or capacity; and the availability, accuracy, use, or knowledge of disease risk models or prediction tools (Table C in S1 Appendix).

*“There is data quality issues due to underreporting and overreporting of cases which will result in unacceptance of the result by the policy makers since it seems inaccurate when tested with actual data, because model doesn’t detect if you feed inflated data. The other challenges are getting the data in time to predict, retaining and attracting manpower to do mathematical modelling, AI modelling, and statistical modelling given the low payment structure.”* [Ethiopia: P4]

The challenge of financial resource limitations was particularly evident amongst KII participants in Somalia (*n* = 13/14, 92.9%), manifesting as funding gaps for disease control, a lack of medical supplies and vaccines, and insufficient capacity amongst healthcare workers.

Amongst FGD participants in Uganda, common challenges in disease preparedness comprised financial and human resource limitations, and sociocultural and behavioural barriers. These included: low literacy rates hindering understanding of public health messages; cultural beliefs associated with specific diseases or meat consumption (described in relation to anthrax risk); and variable acceptance of public health interventions at the community level. Reflecting national-level perspectives, FGD participants in Kampala described policy and system gaps (relating to poor integration of wildlife diseases in policies, lack of standardised tools for outbreak prediction, and inadequate district-level surveillance), in addition to technical and research gaps (in the area of outbreak prediction tool development) and political considerations of disease control strategies and data-sharing (in relation to trade-sensitive diseases).

FGD participants in Somalia highlighted that data deficiencies and poor government preparedness and coordination represented key challenges in disease preparedness.

“[Ministries] *lack proper coordination and do not share information effectively…* [The absence of] *platforms for inter-ministerial coordination and lack of contingency plans prevent an effective response.*” [Somalia: FGD 1, R3]

To mitigate challenges in disease preparedness, the most frequently reported solutions by KII participants (each reported by ≥5) related to: engaging and collaborating with key national stakeholders, sectors, and policymakers (including implementation or institutionalisation of a One Health approach; *n* = 23/63, 36.5%); improving emergency preparedness systems, including national contingency plans and disease-specific early warning systems (*n* = 16/63, 25.4%); improving financial resources and budget allocation (*n* = 17/63, 27.0%); capacity building through implementation of community-level interventions (*n* = 9/63, 14.3%); awareness creation and information dissemination (*n* = 6/63, 9.5%); capacity building in the area of climate-resilient technology (*n* = 5/63, 7.9%); applying evidence- or research- based solutions (*n* = 5/63, 7.9%); and engaging with international collaborators or global experts (*n* = 5/63, 7.9%).

In Uganda, FGD participants in Kampala described the development of disease-specific strategies and guidelines (for RVF, anthrax, and CCHF), targeted community-level interventions (including vaccination campaigns) and provision of technical support, research collaborations via the national One Health platform, and improved surveillance of wildlife health and interfaces. In Kasese, FGD participants described community-focussed solutions, including community sensitisation on health and disease prevention, community-level climate mitigation measures, and development of a district-level climate action plan.

Furthermore, FGD participants in Ethiopia described the need for improved data collection practices amongst field personnel, to enable more accurate disease prediction and timely interventions.

*“Building local capacity in terms of collecting data from a specific location at same time, developing the capacity of grassroots practitioners, especially field epidemiologists at zonal and district level would be of importance in order to predict diseases in a very accurate manner.”* [Ethiopia: FGD 2, B6]

FGD participants in Somalia also re-iterated the need for enhanced disease surveillance and early warning systems, in addition to implementation of community education activities to promote community-level resilience to climate-related diseases.

### 3.3 Disease risk in relation to El Niño-Southern Oscillation and Seasonality

#### 3.3.1 El Niño-Southern Oscillation awareness.

The majority of KII participants and FGD participants at the national level described their understanding of ENSO (or El Niño for the warm phase, or La Niña for the cold phase), with definitions varying depending on stakeholders’ work or geographic context, or individual knowledge level.

*“El Niño and La Niña are natural phenomenon. But with climate change, the severity and the frequency of this phenomena is increasing. El Niño and La [Niña] might happen every five years, but because of the climate change, it can happen every two years. And when it happens, the frequency of drought increases. In Ethiopia, drought used to happen every 10 years, then it comes down to five, and then to two years”* [Ethiopia: P11]

At the district level, awareness of ENSO amongst FGD participants in Kasese, Uganda, was limited, with some individuals believing ENSO to be a political term.

Based on KII participants’ knowledge in their geographic context, the most frequently reported diseases affected by ENSO events (or El Niño or La Niña) were malaria (*n* = 24/63, 38.1%), cholera or diarrhoeal disease (*n* = 20/63, 31.7%), RVF (*n* = 12/63, 19.0%), anthrax (*n* = 9/63, 14.3%), dengue fever (*n* = 7/63, 11.1%) and, more generally, mosquito or vector-borne diseases (*n* = 11/63, 17.5%; Table D in S1 Appendix). These diseases and disease categories were re-iterated by FGD participants in Ethiopia, Somalia and Uganda, with FGD participants in Somalia highlighting sectoral differences in reported diseases (prioritisation of malaria and dengue fever, and RVF and brucellosis, by public health and animal health stakeholders, respectively). Furthermore, FGD participants in Ethiopia highlighted specific disease risks associated with rainfall and temperature variations.

*“When the Pacific Ocean gets warmer, dry air moves into East Africa, leading to drought. As a result, infectious diseases associated with drought increase. When the ocean is cooler, there is excess rainfall, and the number of rainy seasons increases. This supports sustained malaria transmission—even during the typically dry months of March, April, and May, if it rains, there can be another peak in malaria cases. During drought, water sources become scarce, and people may resort to using contaminated water, which increases the risk of cholera and other waterborne diseases.”* [Ethiopia: FGD 2, B5]

#### 3.3.2 Seasonality consideration.

The majority of KII participants reported that seasonality did factor into routine decisions in their working context (*n* = 47/63, 74.6%) and highlighted the importance of considering infectious disease risk during or immediately after the rainy season, stating increased risks of malaria, RVF, cholera, typhoid fever, anthrax (early rainy season), pneumonia, and more generally, waterborne and vector-borne diseases. Fewer KII participants (*n* = 15/63, 23.8%) considered the dry season in relation to disease risk, with participants describing increased risks of drought-associated diarrhoeal diseases and mosquito and tick-borne diseases in relation to poor water management and hot humid weather conditions, respectively.

*“Each disease has its dynamics, e.g., anthrax usually happens when grass is sprouting during the beginning of the rainy season.”* [Uganda: P7]

*“Ticks are more likely to thrive and actively seek hosts in dry seasons due to reduced vegetation, which forces them to move and search for hosts. This leads to an increased risk of transmission of tick-borne diseases during these periods”* [Uganda: P3]

A minority of KII participants (*n* = 5/63, 7.9%) also reported increased respiratory diseases related to periods of increased dust and cold seasons.

*“During the rain season, and also the January, February, March, whereby there is a lot of dust, we can find the asthmatic attacks and also the colds and the flus. Then there's this season of the JJA, that is June, July, August, whereby it's a cold season, we find also the cold related diseases, …the MAM, the March, April, May, where we get the rains together with the OND, that is October, November, December. So we can say the four, are the most… like the waterborne diseases, the cold related diseases, and also the dust related diseases.”* [Kenya: P2]

The strong influence of seasonality on disease patterns and routine decision-making was re-iterated by FGD participants in Uganda, including use of an established local seasonal calendar and association of dry and rainy seasons with specific diseases and vector life cycles. Examples of increased burdens of RVF and anthrax following flooding and land disturbance were described, respectively.

### 3.4 Decision-making processes

#### 3.4.1 Institutional level of decision-making and communicating decisions on future disease risk.

The majority of KII participants reported that decisions related to climate-sensitive disease surveillance and control were made at the central level (*n* = 35/63, 55.6%), encompassing ministry, institution, department, and directorate levels (Table E in S1 Appendix). Stakeholders also reported decision-making at variable sub-national levels (*n* = 6/63, 9.5%) and at the level of programmes or projects (*n* = 4/63, 6.3%).

At the central level, KII participants reported that decision-makers were affiliated with a range of government departments and institutions, including those related to disease surveillance and prevention; epidemiology; public, animal or environmental health; health promotion and education; and meteorology. Furthermore, multiple departments contributed to decisions.

*“Decisions are not made by one department. All actors should be involved in decision making regarding the diseases risks. Decisions are not compartmentalized; it depends on the kind of decision you are going to make. For surveillance; the Department of Integrated Epidemiology, surveillance, and Public Health Emergencies. About Risk Communication; Department of Health Promotion and Education. Regarding diseases like Cholera, the Department of Environmental Health”* [Uganda: P8]

This multi-stakeholder approach was re-iterated by FGD participants in Ethiopia, Somalia, and Uganda, who described the main government institutions responsible for decision-making, with individual contributions varying, depending on the disease or type of climate data under focus. For example:

*“In Ethiopia, the institutional framework for decision making process issues lies in between 5 institutions -the National Meteorological Agency responsible for collecting data and disseminating the meteorological and climate data, the Ministry of Health for overseeing the surveillance system, EPHI* [Ethiopian Public Health Institute] *for taking central role in this surveillance and others, and the national disease Risk Management Commission focus on disaster preparedness in the regional and governmental local governments for decentralized issues. And usually, the decision lies in between this in paper at least, but from the fragmented involvement, as we had previously the framework is here, but it looks a little bit loose.”* [Ethiopia: FGD 2, B1]

In Somalia, FGD participants also described the contribution of multi-lateral agency partners (including the WHO and FAO) to national “cluster meetings” to inform decisions related to outbreak responses.

#### 3.4.2 Relationship between national and sub-national decision-making.

KII participants reported dynamic and variable relationships between national and sub-national decision-making on climate-sensitive disease surveillance and control, and outbreak response. Country-level variations in decision-making pathways, challenges, and solutions, are described in Table F in S1 Appendix.

Reported challenges of these relationships included poor coordination or communication between stakeholders, and variable capacity for decision-making between actors (related to variable support systems, resource availability, and technical expertise). FGD participants in Uganda described the communication flow of central government decisions to district-level personnel for local implementation, and the associated challenges, including disconnect or delays between high-level decision-making and field implementation (associated with resource constraints or inefficient bureaucratic processes).

*“I am looking at decisions made from top to down and then decisions made from bottom to up, I think for better control of some of these diseases we need also to be mindful of decisions that are made at the lower level because that is where implementation and control of some of these diseases occur.”* [Uganda: FGD Kampala, R3]

Additional solutions reported by KII participants included improving cross-sectoral and multidisciplinary collaboration, coordination and communication between stakeholders (for example, through raised awareness of national and sub-national climate and One Health units), availability of advanced tools for informed decision-making, promoting harmonised working conditions, and increasing support and availability of trained personnel at all organisation levels.

In Somalia, FGD participants re-iterated the significant challenge of weak coordination mechanisms between ministries dedicated to human, animal, and crop health, which operate in distinct siloes. In response, an ongoing solution has been the development of a climate change policy to address the needs of each health sector and promote a more integrated One Health approach to decision-making related to climate risks.

### 3.5 Existing data to support decision-making

#### 3.5.1 Data types and sources to inform future disease risk.

KII participants reported a range of data types informing institutional decision-making about future disease risks, including health system data (*n* = 20/63, 31.7%), climate or environmental data (*n* = 14/63, 22.2%), epidemiological data (*n* = 11/63, 17.5%), meteorological or weather data (*n* = 10/63, 15.9%), and disease surveillance data (*n* = 8/63, 12.7%; Fig 2). Disease-specific information collected for decision-making processes included case and mortality statistics, spatial and temporal data, demographic data, information on disease control and interventions, entomological or vector data, and information about the causative agent or strain.

**Fig 2:**
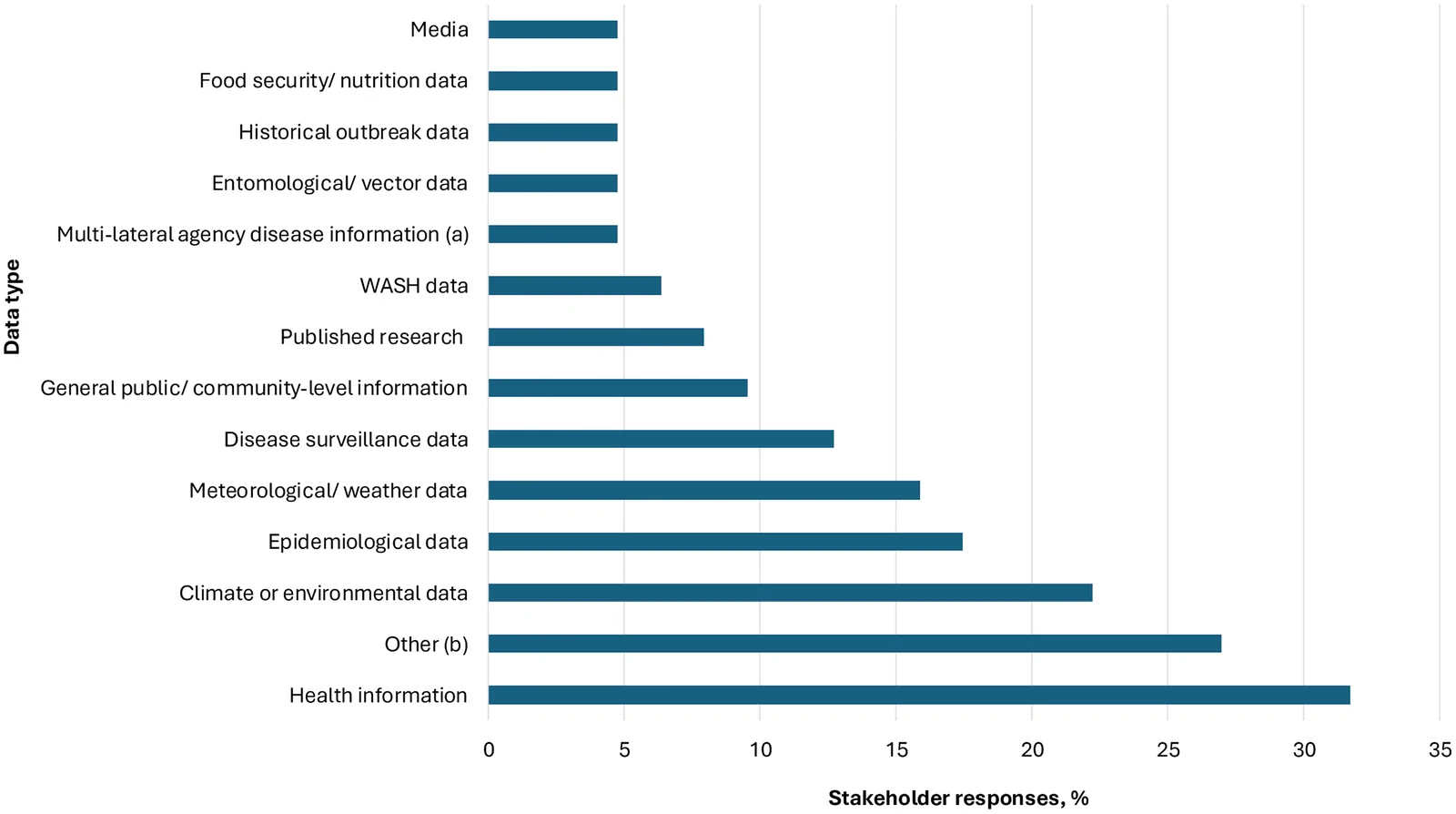
Data types collected for institutional decision-making about future disease risks, as reported by Key Informant Interview participants (*N* = 63). Acronyms: WASH = water, sanitation and hygiene. (a) Disease information from the World Organisation for Animal Health (WOAH), the Food and Agricultural Organization (FAO), and the World Health Organization (WHO). (b) For the following data types: Regional or global information (*n* = 5); organisational report or data (NGO, *n* = 2); human or animal population movement data (*n* = 1); laboratory data (*n* = 1); disaster risk data (*n* = 1); veterinary data (*n* = 1).

Data were sourced predominately from health facilities (*n* = 23/63, 36.5%), the community or community-based organisations (*n* = 13/63, 20.6%), government ministries or institutions (*n* = 11/63, 17.5%), and surveillance personnel or systems (*n* = 7/63, 11.1%; Table G in S1 Appendix). Other data sources (each reported by ≤4 participants) included satellite or meteorology data sources, environment or weather stations, the media (newspapers or social media), researchers or expert opinion, international partners, and an NGO.

Overall, KII participants reported variable integration of climate or disease data to support decision-making in their work environment (integration: *n* = 24/63, 38.1%; no integration: *n* = 28/63, 44.4%). Country-level variations were demonstrated, with participants in Uganda (*n* = 9/16, 56.3%) and Kenya (*n* = 9/21, 42.9%) reporting proportionally higher levels of integration of climate or disease data, compared to Somalia (*n* = 4/14, 28.6%) and Ethiopia (2/12, 16.7%).

#### 3.5.2 Climate or environmental data uses and sources.

The majority of KII participants reported using climate or environmental data in relation to disease risk, with country-level variations demonstrated across Kenya (*n* = 20/21, 95.2%), Somalia (*n* = 9/14, 64.3%), Uganda (*n* = 12/16, 75.0%), and Ethiopia (*n* = 7/12, 58.3%). Overall, the most frequently reported weather or climate factors monitored or used by stakeholders’ organisations were rainfall or precipitation (*n* = 46/63, 73.0%), temperature (*n* = 35/63, 55.6%), and humidity (*n* = 20/63, 31.7%; Table 4). Amongst KII and FGD participants in Ethiopia and Uganda, selection of climate variables was informed by specific disease risks, including cholera, anthrax, and vector-borne disease risks.

**Table 4:** (A) Climate or environmental factors, and (B) climate or environmental data sources, used or monitored by organisations to guide potential risk, reported by Key Informant Interview participants (*N* = 63).

	Stakeholder reports, *n* (%), *N* = 63
(A) Climate or Environmental Factors	
Rainfall or precipitation	46 (73.0)
Temperature	35 (55.6)
Humidity	20 (31.7)
Wind speed, direction, or strength	9 (14.3)
Vegetation cover	5 (7.9)
Solar radiation or sunshine	4 (6.3)
Air pollution	3 (4.8)
Evaporation	3 (4.8)
Flooding	3 (4.8)
Seasonal patterns or forecast (including MAM and OND)	3 (4.8)
Water level	2 (3.2)
Other ^a^	8 (12.7)
**(B) Climate or Environmental Data Sources**	
Government	
Meteorology	25 (39.7)
Environment/ Climate	17 (27.0)
Agriculture	6 (9.5)
Health	5 (7.9)
Disaster	3 (4.8)
One Health	1 (1.6)
Office of Prime Minister	1 (1.6)
Government of another country	3 (4.8)
Multi-lateral agency	10 (15.9)
Satellite website	3 (4.8)
Community reports	3 (4.8)
Academia or research institution	2 (3.2)
Other ^b^	9 (14.3)

Acronyms: MAM = March-April-May forecast; OND = October-November-December forecast; NGO = non-governmental organisation. ^a^For the following factors, *n* = 1 (frequency exceeds total frequency stated in Table 4 as individual participants reported multiple factors): Soil type; area covered by extreme climate events; crop yield; water availability; pasture accessibility; ozone data; extreme weather events (not specified); dust in atmosphere; clouds; topography. ^b^For the following data sources, *n* = 1: Non-governmental organisation; media; malaria project; sentinel sites; climate/ weather station; presentations; online data; briefs; websites with aggregated weather information; international organisation websites; literature review.

*“There are different climate parameters we are currently using such as temperature, relative humidity, rainfall, vegetation index, and land surface temperature which have contributions to parasite survival suitable for mosquito breeding.”* [Ethiopia: P5]

A minority of KII participants reported not using (*n* = 7/63, 11.1%) or limited use of (*n* = 3/63, 4.8%) climate or environmental data due to limited technical expertise or this not being the primary focus of their organisation. In Somalia, KII participants also reported use of climate data was prevented by weak understanding of application in health planning, lack of forecasting tools, poor integration between climate and health systems, absence of formal links with meteorological agencies, and data availability and quality issues.

Sources of climate or environmental data reported by KII participants comprised predominantly government ministries or institutions, of which the majority represented meteorological (*n* = 25/63, 39.7%) or environmental or climate units or departments (*n* = 17/63, 27.0%; Table 4). Other data sources included multi-lateral agencies, satellite data websites, community reports, and academia or research institutions. Use of community-level knowledge on local rainfall patterns and flood risks was described by FGD participants in Kasese, Uganda.

### 3.6 Interventions and strategies informed by existing data

#### 3.6.1 Examples and outcomes of decisions on future disease risk.

KII participants provided examples of specific actions which influenced or managed disease risk, including decisions related to climate-sensitive diseases RVF, malaria, cholera, anthrax, typhoid fever, dengue fever, and yellow fever. Examples of decisions or actions included the following: development of disease contingency plans; vaccination campaigns (including in identified cholera ‘hotspot’ areas, and in areas endemic for yellow fever and dengue fever); vector control (associated with RVF, malaria, and yellow fever surveillance); development of early warning systems and disease outbreak projections; increased multi-stakeholder engagement; government communications and press statements on disease risk (to increase public awareness); disease-specific preparedness strategies (including provision of mosquito nets and anti-malarial treatments, and equipping health facilities); establishing mitigation plans for extreme climate events; and budget allocation.

50.8% of KII participants (*n* = 32/63) reported that these actions led to a positive outcome, which included the following: a reduction in disease burden, mortalities, or outbreaks; amendments to disease control and prevention strategies; the development of policy briefs or improved policy translation; increased community-level education about diseases; improved case management; improved stakeholder collaboration; improved disease preparedness or risk management; and a change to outbreak dynamics.

*“[We] supported the national government develop a contingency plan for Rift Valley fever, mapped the risk of these three diseases, brucellosis, Rift Valley fever and Q fever, worked with the* [removed name] *county government, especially the veterinary department, to vaccinate animals for Rift Valley fever when we learnt that we were going to have heavy rainfall, and there were indications that Rift Valley fever outbreaks was likely to occur. So we worked and supported them to get prepared, and in the end, the impact was minimal.*

*… the collaborative work done with* [removed name] *County government helped to cushion the impacts of a potential outbreak that would have probably caused more damage to the or more losses to the farmers and to the county.”* [Kenya: P9]

*“One specific example is the decision to implement early warning systems and community-based surveillance for cholera outbreaks in Somaliland, influenced by climate variability and flooding risks. This decision, coupled with effective communication campaigns about hygiene practices and clean water use, significantly reduced cholera cases in high-risk areas. By proactively addressing the link between climate conditions and disease spread, the Ministry of Health and Development minimized the impact of future outbreaks and strengthened community resilience.”* [Somalia: P12]

Other positive outcomes (each described by one KII participant), included data model generation, identification of climate risk factors (in relation to vector-borne diseases), improved intervention strategies (for malaria), increased research activities, and increased vaccine production.

FGD participants in Kampala, Uganda, described positive outcomes of reduced anthrax outbreaks and spread following a proactive anthrax vaccination strategy (based on meteorological reports of heavy rain predictions and historical outbreak patterns) and improved One Health implementation and cross-sectoral policy enforcement.

In Somalia, FGD participants described positive outcomes linked to coordinated early warning systems and proactive public health campaigns. Examples included: district-level disaster committees responding rapidly to cholera and measles outbreaks; flood and drought early warning systems, reducing the impact of water-borne diseases; and national campaigns to distribute mosquito nets to reduce malaria cases.

KII participants reporting negative (*n* = 3/63) and mixed outcomes (*n* = 10/63) were situated in Uganda, and in Kenya, Somalia, and Uganda, respectively. These outcomes related to the financial impacts of outbreak-related trade restrictions on livestock and meat products (as reported by the KII participants in Uganda who reported negative outcomes), inaccurate outbreak predictions and government communications, lack of action at the central level, and poor evidence translation to policy.

*“In 2017, there was a suspected outbreak of highly pathogenic Avian Influenza, somehow communication went out before further and serious analysis of the matter and this cost the country a lot because the trade bans were put the country, i.e., poultry products, eggs could not be exported to Kenya and this cost the country a lot to clear the situation. … In the end, when samples were taken to laboratories, they realized that the type was not highly dangerous to humans*.*”* [Uganda: P7]

### 3.7 Existing tools to support decision-making

#### 3.7.1 Digital tool interaction and device use.

More than one third (*n* = 19/51, 37.3%) of KII participants in Kenya, Somalia and Uganda reported interacting with a digital tool or web interface to obtain better information about future disease or climate risk. Stakeholders reported using computers (*n* = 19/63, 30.2%), mobiles (*n* = 18/63, 28.6%), and tablets (*n* = 13/63, 20.6%), and 33.3% (*n* = 21/63) reported using multiple device types. FGD participants in Uganda also reported that the choice of device depended on the tool being used and the volume of data.

#### 3.7.2 Reporting systems in outbreak response.

The majority of KII participants reported using either electronic (*n* = 18/63, 28.6%) or mixed electronic and paper-based systems (*n* = 35/63, 55.6%) to assist decisions in climate-sensitive disease risks in relation to deployment of interventions or outbreak responses. Comparably higher proportions of KII participants in Kenya (*n* = 9/21, 42.9%) and Ethiopia (*n* = 5/12, 41.7%) reported using purely electronic systems, than participants in Somalia (*n* = 2/14, 14.3%) or Uganda (*n* = 2/16, 12.5%).

Strengths of electronic systems for disease surveillance and control, as reported by KII participants, included capacity for faster or real-time reporting to enable rapid decision-making, ability to share data or disseminate information more widely to the target audience, and improved accessibility (including via mobile devices; Table H in S1 Appendix). Reported weaknesses included requirements for a mobile device and Internet or electricity access, lack of data uniformity, resource requirements, and the technological skills or training required.

For KII participants that reported using paper-based systems, reported strengths included ease of use, low cost, and no reliance on electricity or Internet access (Table H in S1 Appendix). Reported weaknesses included the time requirements, unavailability of back-up files, and physical storage issues. FGD participants in Somalia and Uganda (particularly Kasese) reported that paper-based systems were still dominant in rural areas or at the level of the health facility, and that challenges exist in transitioning to fully digitalised systems in resource-constrained settings with limited infrastructure.

#### 3.7.3 Digital tool use.

KII participants (*n* = 31/63, 49.2%) specified digital tools which have been integrated into decision-making processes. These included District Health Information Software (DHIS2), Open Data Kit (ODK), FAO Event Mobile Application (EMA-i+), electronic Integrated Disease Surveillance and Response system (e-IDSR), Epicollect, Kobo Toolbox, and Kenya Animal Bio-surveillance system (KABS). Data collection platforms (including Microsoft Excel-based systems), and generation of summary reports (describing disease surveillance and health register data), were also reported. KII participants reported that use of and access to specific tools within reporting systems varied across the country.

*“There is no uniform reporting system, sometimes they report by phone, sometimes using excel, and other times through emails, because they have to report these notifiable diseases. In Addis Ababa, some reportable diseases are directly entered to DHIS2, but its coverage is not full. There was a direction to use DHIS2 and avoid phone calls, excel sheet, and emails. There are some improvements and now we are working intensively to integrate reportable diseases into DHIS2.”* [Ethiopia: P3]

A range of digital tools were described by FGD participants in Uganda, depending on the government sector (including DHIS2 and EMA-i+ by ministries dedicated to health and agriculture, respectively).

More than half of KII participants (*n* = 35/63, 55.6%) reported that access to digital tool data outputs was institution based or restricted (access rights required or available on request). FGD participants in Uganda re-iterated the restricted access to existing tools and data outputs, due to data sensitivity and the need for specific user rights. Amongst district-level participants in Kasese, tools were perceived as urban-centric, with a requirement for technical skills which may be lacking both amongst health personnel and in the general population. In Somalia, FGD participants described the substantial training required and the major gap in the “digital readiness” of institutions working on climate-sensitive disease risks. Unreliable infrastructure and Internet, limited technical capacity, and systemic issues (such as fragmented data systems and security concerns), were described as drivers of this digital deficit.

In relation to the described digital tools, training requirements or needs described by KII participants related to data management (including collection, input, analysis, access, storage, and reporting), bulletin or report production, and general digital tool or data platform access and use. A minority of stakeholders reported that training needs were dependent on disease event, data under analysis, or individual information technology skills.

#### 3.7.4 Use of modelled output.

One third of KII participants (*n* = 17/51, 33.3%) in Kenya, Somalia and Uganda reported using a modelled output, although a significantly higher proportion of participants reported using modelled outputs in Kenya (*n* = 12/21, 57.1%), compared to Uganda (*n* = 4/16, 25.0%) and Somalia (*n* = 1/14, 7.1%). Of those who reported using modelled outputs, outputs related to the following models and tools were specified: the FAO RVF Early Warning Decision Support Tool (DST); the WHO Early Warning and Response System (EWARS) tools; an autoregressive integrated moving average (ARIMA) model; negative binomial regression models; a machine learning model; geospatial and spatiotemporal models; and weather forecast models. Participants described using disease prediction models for yellow fever, RVF, schistosomiasis, and malaria.

In line with KII outcomes, FGD participants in Somalia re-iterated that there is little use of predictive or analytical climate and/or disease models, with decision-making relying instead on direct observation, historical records, short-term seasonal outlooks, and global platforms (such as WHO’s disease tracking systems).

FGD participants in Uganda highlighted that the ability to perform risk modelling was based on individual competence, that capacity building was required, and that there was no institutionalised model. In rural Kasese, participants described low uptake of modelled outputs, with even basic seasonal forecast information not being effectively utilised.

*“…it means that the weather forecast information we have been receiving has not been utilised by the relevant offices such as DHO* [District Health Office]*... So we agree that modelling information is still lacking.”* [Uganda: Kasese FGD, P3]

Of KII participants that reported *not* using modelled outputs, the majority would consider using them in decision-making on future disease risk.

*“We will be very much ready, because you see, this is an environment body that is supposed to interpret various environmental conditions and how they affect the public. So any model that can make The institution to better interpret such kind of information, the climate change related information, and how this one is likely to affect the public is highly welcome. It will make our work easier, to make us more efficient and also relevant in the areas of our work.”* [Kenya: P6]

These participants elaborated further that future use of modelled outputs would depend on model accuracy, on the availability and input of local data, on validation of the model, and on the model supporting forecasting and decision-making.

### 3.8 Digital tool format and presentation preferences

#### 3.8.1 Digital tool format preferences.

KII participants’ preferences for analysing, displaying, and reporting data for decisions on future disease risks are summarised in Table 5. Preferences for data analysis included options to download data into Excel and perform spatial and quantitative analyses. The most frequently reported preference for displaying *and* reporting data was provision of data summaries and visualisations, including bar graphs, pie charts, line graphs (to show temporal trends), tables, and infographics. For reporting data, stakeholders also reported preferences for data summaries, such as in a report or tabular format or as an executive summary. A preference for a dashboard format was reported by stakeholders for data analysis, display, *and* reporting.

**Table 5:** Stakeholder preferences for data analysis, display, and reporting, as reported by Key Informant Interview participants (*N* = 63).

Analysing	Displaying	Reporting
• Export data into Excel (*n* = 5) • Dashboard format (*n* = 4) • Map format/ spatial analysis (*n* = 4) • Quantitative analysis (including descriptive statistics; *n* = 3) • Tabular format (*n* = 3) • Graph format (*n* = 3) • Offline function (*n* = 1) • Simple data input (*n* = 1)	• Data summaries and visualisations using tables, graphs, charts, and plots (*n* = 25) • Maps (*n* = 12) • Dashboard format (*n* = 5) • Non-aggregated *and* aggregated data (including outbreak- or population-level data; *n* = 2) • Additional information and support links related to displayed data (*n* = 1) • Customise data displayed (*n* = 1) • All interactive elements accessible (*n* = 1)	• Data visualisations using graphs, charts, and infographics (*n* = 9) • Summary in report (Word or PDF file) or tabular format (including data, interpretation, and recommendations; *n* = 7) • Executive summary (*n* = 2) • Dashboard format (*n* = 3) • Digital format (*n* = 2) • Web pages (*n* = 2) • Maps (*n* = 1) • Analysis outputs to inform decision-making (*n* = 1) • Link to information available to all stakeholders (*n* = 1) • Data easy to interpret (*n* = 1) • Appropriate size report (*n* = 1)

These preferences were re-iterated by FGD participants in Uganda who reported the use of Microsoft Excel for analysis, although choice of digital data platform may vary depending on individual expertise or institutional preference. A mixture of text, graphs, and maps were preferred for displaying and reporting data, with participants sharing experiences of shifting from text- to map-based forecasts. In relation to reporting data, the need for compatibility between tools used by different countries was highlighted.

Overall, KII participants reported that visual aids added value to decision-making in relation to future disease risk, in relation to being easier to understand and interpret by, and communicate to, a wider audience (including non-technical decision-makers and funders). Specifically, maps provided value by communicating geospatial information (including outbreak location, disease hot spots, risk maps, and national-level data), whereas graphs enabled analysis of trends (historical, current, and future).

*“If the data are presented spatially, using a map is preferred because visuals can convey more, especially when accompanied by a legend. If hotspot areas are identified, showing which areas are at high or low risk, it is helpful to extract and list the specific woredas and zones alongside the map. The map itself can be explanatory; for example, if a forecast is displayed, anyone can easily understand which areas are at high, moderate, or low risk for malaria by referring to the legend. However, the map alone is not sufficient for decision-making. Therefore, presenting detailed information by extracting and listing high-risk areas at the district level is essential.”* [Ethiopia: P5]

*“They can be understood by the people in decision-making who are not as technical as we are; Use of the technical terms may not be appreciated more by the decision-makers. When we were in a meeting with the WHO, participants were able to highlight that the issues were distributed according to the geography like along the borders and the specific districts, people appreciate more …the visual aids compared to the words.”* [Uganda: P8]

The high value of visuals aids was re-iterated across all FGDs, in relation to providing effective summarisation and presentation of disease data to decision-makers, non-technical audiences, and communities. Participants described the use of visual aids for public health messaging in relation to malaria prevention, maps to highlight malaria, anthrax, and cholera hotspot areas to enhance decision-making, and graphs to indicate temporal and seasonal disease trends.

*“The spatial description should display hotspot areas, and that data should be accessible. The data display method could be in graph or table because it is easily understandable.”* [Ethiopia: FGD 1, A4]

*“Maps and graphs make data easier to interpret, especially for non-technical stakeholders.”* [Somalia: FGD 1, R6]

#### 3.8.2 Device type preferences.

In relation to development of a new digital tool to forecast climate-sensitive disease risk, KII participants described variable preferences for device type, including computers (*n* = 17/63, 27.0%), mobiles (*n* = 34/63, 54.0%), and tablets (*n* = 14/63, 22.2%). About half of the participants (*n* = 32/63, 50.8%) reported a preference for multiple device types.

Preferences for a mixture of device types and for digital tools compatible with multiple device types were re-iterated by FGD participants in Ethiopia and Uganda, although device choice was reported to be influenced by user role, resource availability, volume of data, data analysis requirements, practicality or portability in the field setting, and offline capability.

*“I suggest a mixed-device compatible tool. It may depend on the context, for stationed people, using a computer or tablet might be preferable. However, for high-level decision-makers, who often have tight schedules and are in meetings, accessing information quickly on a mobile phone could be more practical. If I had to choose one, I would say mobile phones might be preferable, assuming everyone has access to smartphones, and they are more readily available and convenient for quick reference. I recommend considering different devices based on varying levels of engagement and decision-making roles.”* [Ethiopia: FGD 2, B5]

*“If you are in the field collecting data, then you need a tablet or a phone. But for us, when you are downloading other data, then you need a machine that can support that.”* [Uganda: Kampala FGD, R6]

#### 3.8.3 Geographical resolution.

KII participants described variable preferences for the geographical resolution of the tool and integrated maps to support decision-making and communicate responses. By country, most participants reported a preference for the geographic resolution to be at the sub-national level, although at variable administrative levels. Due to variations in the terminology and characteristics of administrative divisions between contexts, **Table 6** summarises the preferred geographical resolutions by country.

**Table 6:** Stakeholder preferences for digital tool geographic resolution, reported by Key Informant Interview participants (*N* = 63).

Country	Geographic resolution ^a^	Current administrative divisions (ordered largest to smallest)
**Ethiopia**	• District or *woreda* (*n* = 3) • Sub-district or *kebele* (*n* = 6)	• Region • Zone • *Woreda* (synonymous with district) • *Kebele*
**Kenya**	• National (*n* = 1) • County (*n* = 1) • Sub-county or district ^b^ (*n* = 4) • Ward (*n* = 5) • Sub-location ^b^ (*n* = 1) • Community, village, or lowest level (*n* = 5) • Population catchment area (*n* = 1)	• County • Sub-county • Ward • Village
**Somalia**	• Region (*n* = 1) • District (*n* = 7) • Sub-district (*n* = 1) • Community or village (*n* = 3)	• Region • District
**Uganda**	• State (*n* = 1) • District (*n* = 4) • Sub-district (*n* = 1) • Sub-county (*n* = 8) • Parish (*n* = 2)	• Region • District • County/ City • Sub-county/ Municipality • Parish/ Ward • Village/ Cell

^a^For participants who specified multiple administrative divisions, the smallest division specified is presented; ^b^ District and sub-locations were administrative divisions under the previous constitution (prior to The Constitution of Kenya, 2010).

Preferences for sub-national level geographic resolution were re-iterated by FGD participants in Ethiopia, Somalia, and Uganda, encompassing preferences for data at various levels of administrative division for both countries. In Ethiopia, whilst many participants highlighted that displaying data at the smallest administrative unit would allow more targeted interventions, participants acknowledged that higher district-level resolution might represent the most feasible and practical option, with regards to data availability and actionable interventions. Preferred resolution might also vary depending on specific disease and the intended tool user.

*“When we go lower there is feasibility issue and as we go upper there is data accuracy issue. The app* [application] *can use Eco epidemiological settings because that will enable it to predict for similar eco epidemiological settings since the disease occurrence pattern will also be the same.”* [Ethiopia: FGD 1, A2]

#### 3.8.4 Level of digital tool interaction.

In relation to level of tool interaction, KII participants reported the following tool characteristics which may optimise decisions and communications regarding future disease risks (each reported by ≥3 participants): a data download or export function, to export raw data, summaries, or visualisations; incorporation of or ability to generate data visualisations; being user-friendly and practical to use; availability of sub-national level data; the ability to interact with or generate reports; and the ability to make disease predictions. Additional suggestions included incorporating a zoom function, a function for tool users to comment, ability to use offline, limiting access to users based on position, provision of information on outbreak magnitude (to inform preparatory measures), integration of climate or environmental data, ability to filter analysis by disease or time period, and flexibility to incorporate datasets from different sources.

*“The level of user interaction with the system should be limited based on their position, as it would be challenging if unauthorized users could alter the data. For example, a data entry officer should only have access to enter and edit data, not to modify data after analysis. In our context, where everything is tied to reporting, the system shouldn’t be exposed to manipulation. It is acceptable for users to download it for later use as long as they aren’t manipulating the raw data in the system.”* [Ethiopia: P3]

*“I want to see information on the potential magnitude of the outbreak. Not just state that there is a likelihood for an outbreak. I need to know how much effort should I put in to prepare. Anything that will help me to know the exact place, the exact location.”* [Kenya: P1]

To optimise decision-making regarding future disease risks, FGD participants in Ethiopia and Uganda also emphasised the need for the tool to be user-friendly (including distinct functions for the public versus technical personnel), to produce comprehensive but easily understandable statistical reports, and to have offline functionality.

*“When accessing the app, it should be GPS accessible and special consideration should be given to areas with no connectivity so that they can download when they have internet connection and access the data offline”* [Ethiopia: FGD 1, A1]

In Somalia, one FGD participant also highlighted that the tool should integrate into existing digital health systems and bridge key sectors to avoid either duplication of efforts or the creation of data siloes.

The tool must *“…integrate data from health, agriculture, and climate to provide a holistic picture.”* [Somalia: FGD 1, R1]

### 3.9 Data preferences

#### 3.9.1 Disease data types.

KII participants reported disease data variables that would be useful in advance, which included populations (*n* = 28/63, 44.4%) and geographic areas at risk (*n* = 9/63, 14.3%), and outbreak statistics such as prevalence (*n* = 28/63, 44.4%) and case number (*n* = 21/63, 33.3%; **Table 7**). Comparable proportions of participants between countries reported that prevalence data would be useful, whereas a higher proportion of participants situated in Uganda reported that case number and data on populations at risk would be useful.

**Table 7:** Stakeholder disease data type preferences, reported by Key Informant Interview participants (*N* = 63).

Disease data type	Stakeholder reports, *n* (%), *N* = 63
Disease statistics	
Prevalence	28 (44.4)
Case number	21 (33.3)
Mortality or case fatality rate	6 (9.5)
Incidence	5 (7.9)
Trends over time or historical data	5 (7.9)
Morbidity	2 (3.2)
Attack rate or risk	2 (3.2)
**Population(s) or areas at risk**	
Population(s) ^a^	28 (44.4)
Geographic areas	9 (14.3)
**Risk factors or environmental data**	
Climate or environmental data	5 (7.9)
Vector or entomological data	3 (4.8)
Risk factors	2 (3.2)
Disease characteristics ^b^	2 (3.2)
Population movements	1 (1.6)
**Other**	
Interventions/ management	6 (9.5)
Human livelihood data	2 (3.2)
Economic impact	1 (1.6)

^a^Including response ‘gender’ (*n* = 1); ^b^ Including zoonotic potential, mode of transmission, disease cause, and vectors.

*“The tool should include expected case numbers, the timing of disease occurrence, and the corresponding interventions required at each level. Cases may surpass predefined thresholds, moving through different phases such as the alert phase, outbreak phase, and widespread outbreak phase, each requiring a specific level of response. When case numbers exceed the threshold, there are preparedness and outbreak response activities. Additionally, the tool should include information on the population at risk to support targeted interventions.”* [Ethiopia: P1]

FGD participants in Uganda elaborated on these data variables, highlighting the need for key epidemiological data (including data on disease characteristics, population and geographic areas at risk, season, and demographic characteristics), in addition to data on clinical signs and symptoms.

#### 3.9.2 Optimal lead-time to receive information.

Regarding future disease risks, KII participants most frequently reported optimal lead-times of 1–3 months (*n* = 29/63, 46.0%) to receive information of an impending risk or extreme event so they can deploy intervention strategies (Fig 3). KII and FGD participants also reported that lead-time requirements may vary (*n* = 11/63, 17.5%), for example depending on the disease, outbreak severity, population at risk, geographic area, data availability, or resources.

**Fig 3:**
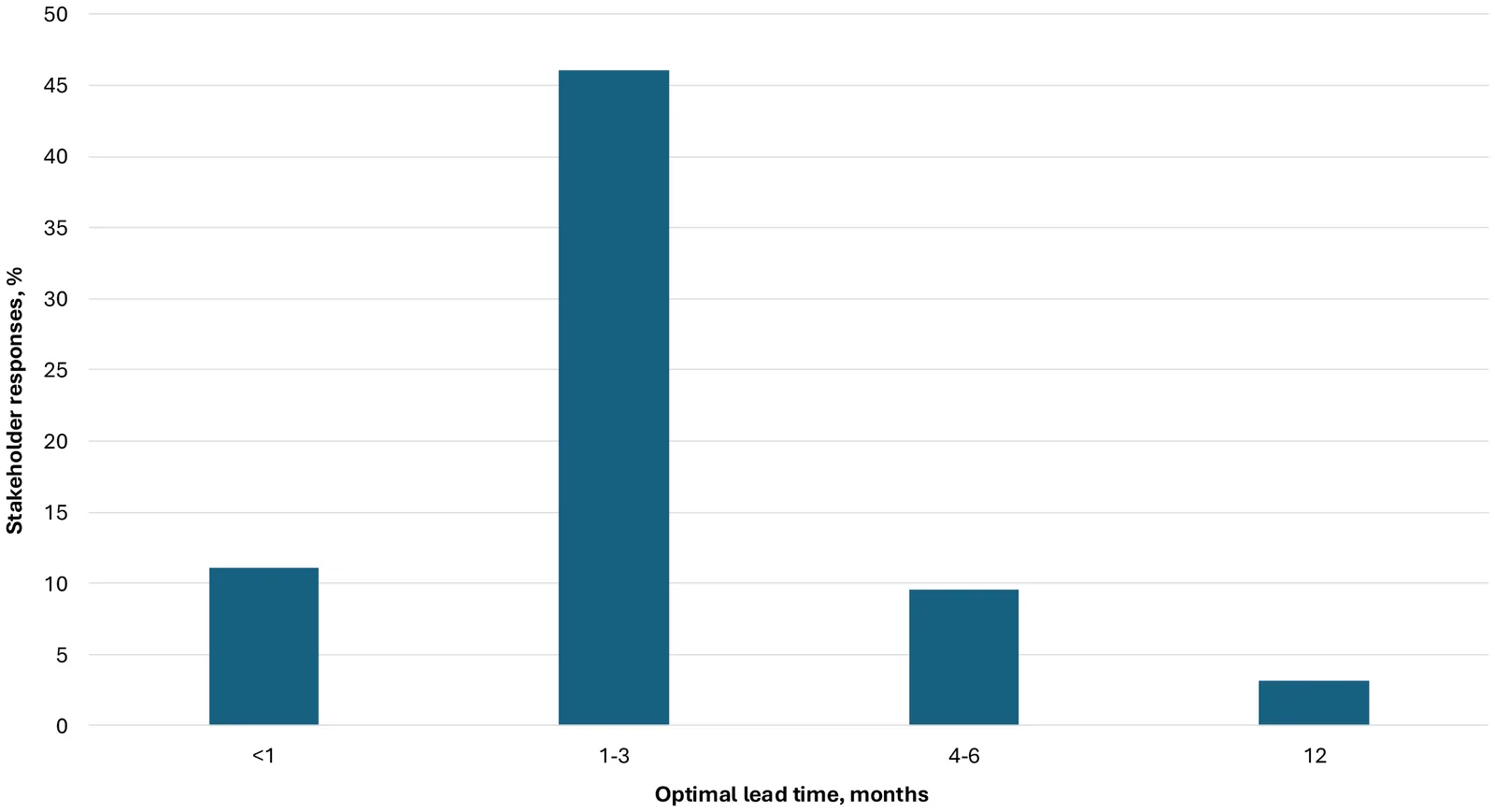
Optimal lead-times to receive information of an impending risk or extreme event, as reported by Key Informant Interview participants (*N* = 63).

Excludes KII participant response ‘varies’ (*n* = 11/63, 17.5%): Encompasses participants who reported variable optimal lead-times (depending on disease, outbreak severity, population at risk, geographic area, data availability, or resources).

*“We can make predictions for a year but that affects the level of accuracy. So, we should make it three months but if we are accurate enough a year ahead is also good to get prepared better and update it at six months and three months.”* [Ethiopia: FGD 1, A5]

FGD participants in Kampala, Uganda, reported optimal lead-times ranging from <1 month for high mortality and rapid-onset diseases, to three months for climate-sensitive diseases, to align with quarterly meteorological forecasts and disease or vector life cycles.

### 3.10 Digital tool sustainability

#### 3.10.1 Barriers.

KII participants reported a range of barriers to sustaining the use and maintenance of a new digital tool, including cost, lack of technological expertise or support, poor offline capacity or Internet access, poor stakeholder engagement and awareness, and training requirements (Table 8).

**Table 8:** Summary of (A) barriers and (B) solutions to digital tool sustainability, as reported by Key Informant Interview participants (*N* = 63).

	Stakeholder reports, *n* (%), *N* = 63
(A) Barriers ^a^	
Cost or not cost-effective	23 (36.5)
Lack of technological skills, expertise, or support	16 (25.4)
Poor offline capacity or Internet access (including related charges)	9 (14.3)
Poor stakeholder engagement and awareness (including poor inclusivity)	8 (12.7)
(Long-term) Training requirements	8 (12.7)
Tool not user-friendly (including ease of data input or export)	7 (11.1)
Long-term tool ownership or availability of host	7 (11.1)
Long-term maintenance	6 (9.5)
Lack of human resources	5 (7.9)
Resistance to new technology or shift from paper-based system	4 (6.3)
Poor data availability or quality	3 (4.8)
Lack of political or government support	3 (4.8)
Poor data security	2 (3.2)
**(B) Solutions** ^**b**^	
Targeting or engaging stakeholders (including the end-users)	19 (30.2)
Capacity building and training	22 (34.9)
Financial resources or funding mobilisation	13 (20.6)
Selecting the correct host or owner ^c^	12 (19.0)
Developing a user-friendly tool (including provision of information on use)	10 (15.9)
Inclusion of stakeholders in tool development or addressing their needs	7 (11.1)
Cost-effectiveness	7 (11.1)
Tool monitoring and maintenance	7 (11.1)
Creation of a dynamic/ flexible tool (including routine reviews or updates)	6 (9.5)
Build tool awareness or tool sensitisation	5 (7.9)
Providing technical support or skills	4 (6.3)
Offline function	4 (6.3)
Availability on mobile device (including app development)	3 (4.8)

^a^Barriers reported by ≥3 participants; additional reported barriers (*n* < 3) included: lack of a business model; lack of data sharing; poor tool acceptance; poor end user engagement or benefit; poor access on a mobile device; conflicting roles or mandates between departments; tool system failures; tool integration challenges. ^b^Solutions reported by ≥3 participants; additional reported barriers (*n* < 3) included: ensuring information is utilised; providing a tool demonstration; providing accurate predictions or adequate sensitivity and specificity; community or grassroots engagement; international partnerships; build infrastructure for data management; assign teams to maintain or operate tool; gradual implementation; defining the user population. ^c^Host or owner suggestions included national government, a regional body, and Africa CDC.

*“The challenges to sustainability of the tool are firstly, there is an infrastructure issue; secondly, lack of manpower. On top of that, continuous training is important because the person responsible changes every time. The other thing is maintaining the finance after the project ends, because there are running costs.”* [Ethiopia: P3]

FGD participants in Somalia and Uganda re-iterated that barriers to tool sustainability included Internet access and connectivity, financial and budgetary constraints, maintenance (in relation to maintaining data quality), and tool governance and ownership issues (with a call for local ownership by participants in Uganda to reduce dependence on external developers for support and updates).

*“…keeping data up-to-date requires a robust reporting system that is not easy to sustain.”* [Somalia: FGD 1, R4]

*“We need a system that speaks to Ugandans... if we have a problem, we come to you* [local expertise]*, we are able to handle it. But if we say, wait a minute, I talk to England people... let it not be a daily thing.”* [Uganda: Kampala FGD, R4]

FGD participants also described a major challenge being integration of new tools into existing national systems (with new tools required to either integrate seamlessly or offer clear compelling advantages), in addition to limited technical capacity, and stakeholder or community uncertainty in the perceived benefit or importance of either inputting data or using tools.

#### 3.10.2 Solutions.

Potential solutions suggested by KII participants to address the barriers related to sustainability included: targeting or engaging stakeholders; provision of capacity building and tool training; funding mobilisation; selecting the correct tool host or owner; development of a user-friendly tool; and inclusion of stakeholders in tool development (Table 8).

*“A lot of consultations need to be done with major stakeholders. The tool must be popularized and made affordable. It should be contextualized to address the challenges specific to Uganda*.” [Uganda: P9]

*“We need to find a way to mainstream the agreed-upon resolutions from conferences and workshops. We should invite the actual technical people from different ministries who are responsible for data and information to share their ideas and technical expertise*.” [Uganda: P8]

FGD participants in Ethiopia, Somalia, and Uganda re-iterated the importance of tools being user-friendly (in relation to ease of installation, use, and maintenance), tools having offline capability, and building end-user capacity and technical skills for tool maintenance and adaptation (including at sub-national levels).

*“The tool should be simple, with a built-in user guide to help users interpret and work with the data. Since people have different levels of tech skills, raising awareness and building a common understanding is essential. It's also important to build capacity at the regional or zonal level and include offline access so users can review data even without internet.”* [Ethiopia: FGD 2, B1]

In relation to barriers to tool sustainability described by FGD participants in Uganda and Somalia, the importance of tool integration with existing systems was re-emphasised which would enable local ownership and strengthen governance and institutional coordination (for example, by establishing a cross-sectoral taskforce to manage the tool). In Uganda, participants highlighted that the value, benefits, and relevance of tools to end-users should be clearly communicated.

## 4. Discussion

### 4.1 Main outcomes

This qualitative study highlights a range of stakeholder perspectives towards climate-sensitive disease priorities and risk, existing data and tools to support decision-making and related gaps, and the needs and preferences for future digital tools. These data will inform the development of the CLIMSEDIS dashboard for priority climate-sensitive diseases in the Horn of Africa [20].

Primary climate-sensitive diseases of concern across all countries were malaria, RVF, cholera, dengue fever, and anthrax, of which malaria, RVF, and cholera were the three primary concerns of predominant stakeholder groups representing government, UN agencies, and non-profit or NGOs. These priorities reflect regional and national climate-sensitive and infectious disease priorities [23–27]. Furthermore, they reflect recognised seasonal variations in the epidemiology of these diseases and the continued upsurge of climate-sensitive infectious disease outbreaks across the Horn of Africa, associated with extreme weather events, human displacement, food insecurity and malnutrition, and limited health care access [6,28]. Closer examination of how stakeholder groups and countries define ‘priority disease’ is warranted, including the disease prioritisation methodologies and criteria used, and the extent to which regional, national, and sub-national level of endemicity are considered. Although stakeholders broadly reported similar climate-sensitive diseases of concern across the study countries, future research could examine the inter- and intra-country variations in disease endemicities, and surveillance, control, and response challenges. Understanding stakeholders’ priorities and preferences will allow existing disease models, such as those for RVF and malaria, to be used for digital tool development [29,30]. Fig 4 summarises the common stakeholder needs across study countries related to disease data, decision systems, and digital tool characteristics.

**Fig 4:**
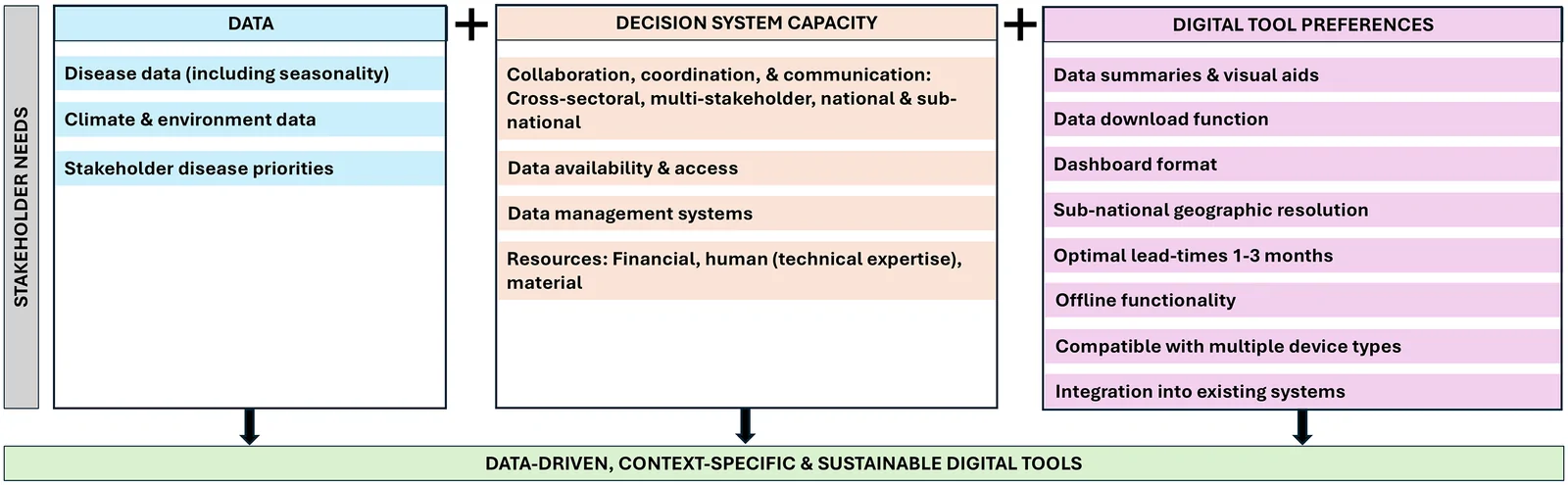
Stakeholder needs related to disease data, decision systems, and digital tool characteristics to inform digital tool development in the Horn of Africa.

Regional- and national-level climate, environmental, and disease data sources can be utilised by climate-sensitive disease forecasting models and may determine the complexity and development of related digital tools; thus, continued examination of current data sources, including access, availability, and application, is critical. Identified country-level variations in the use of climate and disease data to inform decision-making, and related country-specific barriers to data availability and use, may restrict model robustness in certain contexts. Furthermore, inter-country variations in preferences for specific disease data variables could be examined amongst a larger sample size to determine the significance of these variations, and/or whether they reflect the robustness or deficiencies of existing national data systems.

Most stakeholders considered seasonality in routine decisions, although country-level variations were detected in relation to levels of integration and use of climate or disease data to support decision-making. In addition, country-level variations were measured relating to the use of electronic reporting systems, digital tools, and modelled outputs, reflecting variable and uneven levels of digitalisation across the region [31]. Stakeholders reported several constraints, including variable access to mobile devices or Internet, resource limitations, and the technological skills or training required. Those located in more resource-limited settings or working at the sub-national level described their reliance on paper-based systems. For example, in Somalia, participants described challenges in disease preparedness, inefficient paper-based systems, low interaction with relevant digital tools, and an absence of predictive modelling systems. Understanding these challenges will inform tool development and adaptation to variable contexts [32]. Furthermore, country-level variations in the use of modelled outputs, and the reasons provided for variable uptake of models, should be examined further to inform context-specific strategies for sustainable dissemination, integration, and application of the digital tool. For example, in Somalia, reasons why stakeholders prefer to use other information resources should be explored, which might relate to the aforementioned digital deficit of national institutions working on climate-sensitive disease risks.Decision-making related to climate-sensitive disease surveillance and control was described at both central and sub-national levels, highlighting the importance of understanding disease priorities at variable administrative levels and fully engaging local government personnel who may be the primary users of digital tools. The multi-stakeholder nature of decision-making was highlighted across countries, underscoring the importance of a One Health approach, with key stakeholders varying by disease or climate event. Furthermore, variable resource availability and technical capacity were reported to impact stakeholders’ capacity for decision-making. Acquiring an understanding of country-specific decision-making pathways and governance structures is critical in order to target the appropriate stakeholders and primary end-users of a digital tool. In response to evolving and variable health and climate agendas at national, regional, and global levels, continued examination of these decision-making pathways is necessary to direct tool ownership, dissemination, and integration plans.

In relation to preferences for future digital tool format, stakeholders emphasised the value of incorporating a range of data visualisations to effectively summarise and communicate data [33]. Preferences for a dashboard format, with a function to download data, were reported. Across variable contexts, dashboards have been developed to address a range of public health challenges, including infectious (e.g., dengue fever) and non-communicable diseases [34]. Furthermore, participants described a preference for tools with sub-national level geographic resolution.

Infrastructural and resource limitations can significantly hinder tool uptake and sustainability, highlighting the importance of supporting local capacity in conjunction with digital tool development [35,36]. Tool preferences and barriers varied between countries at regional, national, and sub-national levels, re-emphasising the importance of engaging central and sub-national government, in partnership with regional agencies, NGOs, and academia. This was demonstrated by additional assessments by an independent NGO across four counties in Kenya [37], which reinforced our study’s findings including the importance of considering seasonality in relation to disease risk, a strong preference for digital tools with visual aids, and challenges related to digital tool sustainability. Study outcomes have been integrated into the development of the CLIMSEDIS dashboard and updated versions have been assessed during dashboard piloting exercises with key stakeholders. Timely integration of stakeholder feedback at stages of digital tool development, and sustained examination of the needs and priorities of key climate stakeholders across the Horn of Africa, are crucial to ensure the sustainable adoption and integration of the CLIMSEDIS dashboard. Piloting exercises and strategies for long-term tool uptake should be tailored to consider the inter-country variations in local capacity related to data and decision systems.

### 4.2 Limitations

The main limitations included: (i) the unequal number of key informants per country with more KIIs participants and no FGDs conducted in Kenya; (ii) the variable proportions of participants representing different stakeholder affiliations within and between countries; and (iii) the variable rates of non-responses for specific question topics per country. These sample variations reduce the likelihood of thematic saturation being achieved and should be considered when interpreting aggregate figures. Furthermore, purposive sampling technique and the broad scope of participant selection criteria may increase the risk of selection bias. Stakeholders were selected based on the research team’s judgement of their expertise and characteristics of their affiliations (for example, organisations with a focus on public health and climate). Selection bias was likely minimised by the relatively small and interconnected group of organisations and individuals focussing on climate-sensitive infectious diseases in each study country, together with the selection of key stakeholders by researchers who were already situated within target institutions and were therefore, knowledgeable about whom to engage.

Distributions of participant affiliations varied between the countries and should be considered when applying digital tool preferences to inform future digital tool development. Further examination of the extent to which perspectives and digital tool preferences vary by participant affiliation, and between countries for participants with the same affiliation, are warranted. For example, future research could explore if the views of Ethiopian academics are representative of the views of academics elsewhere across the Horn of Africa. Digital tool design should be tailored to the intended primary users and their preferences, which may differ between affiliations and by country.

Due to minor country-level variations in approaches to data collation, which limited data synthesis and deeper qualitative interpretation, descriptive summary statistics were applied to standardise reporting of country-level and aggregated datasets.

Logistical constraints during stakeholder engagement activities included variable stakeholder responsiveness during sensitisation, and challenges in securing appointments with high-level stakeholders (which prevented FGDs from being held in Kenya).

## 5. Conclusion

This study emphasised climate-sensitive disease priorities and related digital tool preferences, amongst key multidisciplinary stakeholders across the Horn of Africa. Country variations in the availability and use of digital tools were identified, reflecting uneven levels of technical capacity and digitalisation across the region, and highlighting a need to support local infrastructure alongside digital tool development and implementation. Local ownership and context-specific approaches would support long-term tool sustainability. These study outcomes will inform the development of sustainable climate-sensitive disease forecasting tools.

## Supporting information

S1 File Interview question guide. Screenshot of CLIMSEDIS dashboard version 1 (pages 6 and 12) available from https://c-tabiri.badili.co.ke/dashboard (access date 21/01/2025). The base layer map was sourced from © OpenStreetMap contributors, available under the Open Database License (ODbL) and from https://www.openstreetmap.org/#map=4/38.00/-95.80 (access date 17/04/2026). Map data attributed to OpenStreetMap contributors (2015) and available from https://www.openstreetmap.org [38]. The shapefiles were sourced from https://data.humdata.org (access date 20/10/2026) [39] and https://open.africa (access date 20/10/2026) [40].
(PDF)

S1 Appendix Supplementary results. **Table A in S1 Appendix.** Countries and organisational affiliations of stakeholders participating in Key Informant Interviews (*N* = 63). Acronyms: NGO = Non-Governmental Organisation ^a^ Including virus research institute (*n* = 1); ^b^ Including virus research institute (*n* = 1); ^c^ Including veterinary research institute (*n* = 1); ^d^ UN agency and government (Planning and Development; *n* = 1); ^e^ UN agency, other multi-lateral agency, and academia (*n* = 1). **Table B in S1 Appendix.** Countries and organisational affiliations of stakeholders participating in Focus Group Discussions (*N* = 57). Acronyms: NGO = non-Governmental Organisation. ^a^ Assistant Professor, lecturer, and researcher positions. **Table C in S1 Appendix.** Challenges in climate-sensitive disease preparedness reported by Key Informant Interview participants (*N* = 63). ^a^ For the following ‘other’ challenges, *n* = 1: Accuracy of notifications; sustainability; trade sensitive nature of disease; vector surveillance; variable preparedness for different diseases; research capacity; not primary focus of organisation; logistical constraints (including access to rural areas). **Table D in S1 Appendix.** Diseases more affected by the El Niño-Southern Oscillation (ENSO) or La Niña events, as reported by Key Informant Interview participants (*N* = 63). Acronyms: CCHF=Crimean Congo Haemorrhagic Fever; RVF=Rift Valley Fever. ^a^ Diseases within these categories not specified. For the following diseases, *n* = 1: ^b^ Dysentery; bluetongue; trypanosomiasis; lumpy skin disease; leishmaniasis; ^c^ Yellow fever; ^d^ Influenza; Marburg; West Nile virus; schistosomiasis; yellow fever. **Table E in S1 Appendix.** Institutional levels of decision-making in relation to climate-sensitive disease surveillance and control, reported by Key Informant Interview participants (*N* = 63). ^a^ Includes stakeholder responses: Central, Ministry, “*institution head*”, and “*top level*”. ^b^ Includes stakeholder responses: Sub-national, district, regional (referring to sub-national administrative unit). ^c^ Includes stakeholder responses: Program, project dependent, and donor dependent. ^d^ NGO (*n*=1) with international and regional (multi-country) offices. **Table F in S1 Appendix.** Relationships between national and sub-national level decision-making on climate-sensitive diseases, as reported by Key Informant Interview and Focus Group Discussion participants. **Table G in S1 Appendix.** Sources of data collected for institutional decision-making about future disease risks, reported by Key Informant Interview participants (*N* = 63). Acronyms: CBO=community-based organisation; NGO=non-governmental organisation. **Table H in S1 Appendix.** Strengths and weaknesses of electronic and paper-based systems for disease surveillance and control, reported by Key Informant Interview participants (*N* = 63). ^a^ Reported by ≥2 participants.
(DOCX)

S1 Checklist COREQ (COnsolidated criteria for REporting Qualitative research) Checklist. (PDF)

## References

[1] Trisos C, Adelekan I, Totin E, Ayanlade A, Efitre J, Gemeda A. Climate Change 2022: Impacts, Adaptation and Vulnerability Contribution of Working Group II to the Sixth Assessment Report of the Intergovernmental Panel on Climate Change. Cambridge, UK and New York, NY, USA: Cambridge University Press; 2022. p. 1285–455. doi: 10.1017/9781009325844.011

[2] Wainwright C, Finney D, Kilavi M, Black E, Marsham J. Extreme rainfall in East Africa, October 2019–January 2020 and context under future climate change. R Meteorol Soc. 2020;76. doi: 10.1002/wea.3824

[3] Alcayna T, Fletcher I, Gibb R, Tremblay L, Funk S, Rao B, et al. Climate-sensitive disease outbreaks in the aftermath of extreme climatic events: A scoping review. One Earth. 2022;5(4):336–50. doi: 10.1016/j.oneear.2022.03.011

[4] UNICEF. Regional call to action - Horn of Africa drought crisis: Climate change is here now. 2022.

[5] World Health Organization. Climate change and health. 2025 [cited 2025 Sep 7]. Available: https://www.who.int/teams/environment-climate-change-and-health/climate-change-and-health/country-support/building-climate-resilient-health-systems/emergency-preparedness-and-management

[6] Norris L, Lemin M, Kelly-Hope LA. Spatial-temporal analysis of natural hazards and disasters in the Greater Horn of Africa between 2010 and 2024 to inform disaster risk reduction, and surveillance and control strategies for climate and environmentally sensitive diseases. BMJ Open. 2025;15(11):e104998. doi: 10.1136/bmjopen-2025-104998 41248381 PMC12587947

[7] Centre for Research on the Epidemiology of Disasters (CRED) - University of Louvain (UCLouvain). EM-DAT - The International Disaster Database. 2023 [cited 2025 Feb 1]. Available: https://www.emdat.be/

[8] FloodList – Floods and flooding news from around the world. [cited 2025 Mar 19]. Available: https://floodlist.com/

[9] Global Flood Awareness System (GloFAS). [cited 2025 Mar 20]. Available: https://global-flood.emergency.copernicus.eu/

[10] East Africa Drought Watch (EADW) - Home. 2025 [cited 2025 March 20]. Available: https://droughtwatch.icpac.net/

[11] Digital Earth Africa. [cited 2025 Marh 20]. Available: https://digitalearthafrica.org/

[12] Copernicus - Climate Data Store. [cited 2025 Mar 20]. Available: https://cds.climate.copernicus.eu/

[13] ECMWF - Open data. [cited 2025 Mar 20]. Available: https://www.ecmwf.int/en/forecasts/datasets/open-data

[14] World Health Organization. COP29 special report on climate change and health: Health is the argument for climate action. Geneva: World Health Organization.; 2024. Available: https://cdn.who.int/media/docs/default-source/environment-climate-change-and-health/58595-who-cop29-special-report_layout_9web.pdf?sfvrsn=dd2b816_8

[15] Thomson MC, Muñoz ÁG, Cousin R, Shumake-Guillemot J. Climate drivers of vector-borne diseases in Africa and their relevance to control programmes. Infect Dis Poverty. 2018;7(1):81. doi: 10.1186/s40249-018-0460-1 30092816 PMC6085673

[16] Bastawrous A, Armstrong MJ. Mobile health use in low- and high-income countries: An overview of the peer-reviewed literature. J R Soc Med. 2013;106(4):130–42. doi: 10.1177/0141076812472620 23564897 PMC3618170

[17] Stanton M, Molineux A, Mackenzie C, Kelly-Hope L. Mobile Technology for empowering health workers in underserved communities: New approaches to facilitate the elimination of neglected tropical diseases. JMIR Public Health Surveill. 2016;2(1):e2. doi: 10.2196/publichealth.5064 27227155 PMC4869228

[18] Digital technologies: A new determinant of health. Lancet Digit Health. 2021;3:e684. doi: 10.1016/S2589-7500(21)00238-7 34711372

[19] Wellcome Trust in collaboration with Inter-American Institute for Global Change Research. Landscape mapping of software tools for climate-sensitive infectious disease modelling. Med Geogr. 2022. Available: https://hetco.io/tools-for-climate-sensitive-diseases/Landscape_mapping_of_software_tools_for_climate_sensitvie_infectious_disease_modelling.pdf

[20] Caminade C, Morse AP, Fevre EM, Mor S, Baylis M, Kelly-Hope L. Forecasting the risk of vector-borne diseases at different time scales: An overview of the CLIMate SEnsitive DISease (CLIMSEDIS) Forecasting Tool project for the Horn of Africa. In: Proceedings of the EGU General Assembly 2023. Vienna: 2023. EGU23-7652. doi: 10.5194/EGUSPHERE-EGU23-7652

[21] Beck HE, Zimmermann NE, McVicar TR, Vergopolan N, Berg A, Wood EF. Data Descriptor: Present and future Köppen-Geiger climate classification maps at 1-km resolution. Sci Data. 2018;5:180214. doi: 10.1038/sdata.2018.214 30375988 PMC6207062

[22] Runfola D, Anderson A, Baier H, Crittenden M, Dowker E, Fuhrig S, et al. GeoBoundaries: A global database of political administrative boundaries. PLoS One. 2020;15(4):e0231866. doi: 10.1371/journal.pone.0231866 32330167 PMC7182183

[23] Bosco Isunju J, Bukenya JN, Nyenje P, Nalugya A. Climate change health vulnerability and adaptation assessment (VAA) for sound management of climate change-related health risks in Uganda. Ministry of Health; 2023.

[24] Ministry of Environment CC and F. National Climate Change Action Plan (NCCAP) III 2023-2027. Nairobi, Kenya; 2023. Available: https://emsi.co.ke/wp-content/uploads/2024/08/Kenya-NCCAP-2023-2027-1.pdf

[25] Vogel M, Alcayna T, Poole L, Baumgart N, Amankona A, Bailey M. Anticipatory Action for Climate-Sensitive Infectious Diseases: East Africa Regional Assessment. 2024. Available: https://www.anticipation-hub.org/Documents/Research_Reports/Anticipatory_action_for_climate_sensitive_infectious_diseases.pdf

[26] Republic of Uganda Ministry of Health. National Technical Guidelines for Integrated Disease Surveillance and Response Third Edition. 2021. Available: https://www.afro.who.int/sites/default/files/2021-09/2_Uganda%203rd%20IDSR%20Tech%20Guideline_PrintVersion_10Sep2021.pdf

[27] The Federal Republic of Somalia Ministry of Environment and Climate Change. Somalia National Climate Change Policy. 2023. Available: https://moecc.gov.so/wp-content/uploads/2024/10/Somalia-National-Climate-Change-Policy-EN.pdf

[28] WHO Regional Office for Africa. The greater Horn of Africa’s climate-related health crisis worsens as disease outbreaks surge [Internet]. World Health Organization African Region. 2022 [cited 2025 Mar 16]. Available: https://www.afro.who.int/news/greater-horn-africas-climate-related-health-crisis-worsens-disease-outbreaks-surge

[29] Chemison A, Ramstein G, Jones A, Morse A, Caminade C. Ability of a dynamical climate sensitive disease model to reproduce historical Rift Valley Fever outbreaks over Africa. Sci Rep. 2024;14(1):3904. doi: 10.1038/s41598-024-53774-x 38365824 PMC10873308

[30] Leedale J, Tompkins AM, Caminade C, Jones AE, Nikulin G, Morse AP. Projecting malaria hazard from climate change in eastern Africa using large ensembles to estimate uncertainty. Geospat Health. 2016;11(1 Suppl):393. doi: 10.4081/gh.2016.393 27063736

[31] Digitalizing Eastern and Southern Africa. [cited 2025 Sep 8]. Available: https://www.worldbank.org/en/region/afr/brief/afe-digital

[32] Mableson HE, Martindale S, Stanton MC, Mackenzie C, Kelly-Hope LA. Community-based field implementation scenarios of a short message service reporting tool for lymphatic filariasis case estimates in Africa and Asia. Mhealth. 2017;3:28. doi: 10.21037/mhealth.2017.06.06 28828375 PMC5547175

[33] Schulze A, Brand F, Geppert J, Böl GF. Digital dashboards visualizing public health data: a systematic review. Front Public Health. 2023;11:999958. doi: 10.3389/fpubh.2023.999958 37213621 PMC10192578

[34] Almasi S, Bahaadinbeigy K, Ahmadi H, Sohrabei S, Rabiei R. Usability evaluation of dashboards: A systematic literature review of tools. Biomed Res Int. 2023;2023:9990933. doi: 10.1155/2023/9990933 36874923 PMC9977530

[35] Manyazewal T, Woldeamanuel Y, Blumberg HM, Fekadu A, Marconi VC. The potential use of digital health technologies in the African context: A systematic review of evidence from Ethiopia. NPJ Digit Med. 2021;4(1):125. doi: 10.1038/s41746-021-00487-4 34404895 PMC8371011

[36] Getachew E, Woldeamanuel Y, Manyazewal T. Capacity and readiness assessment of healthcare facilities for digital health interventions against tuberculosis and HIV in Addis Ababa, Ethiopia. Front Digit Health. 2022;4:821390. doi: 10.3389/FDGTH.2022.821390 PMC8918491 35295619

[37] Save the Children. Climate-Sensitive Disease Tool in the Horn of Africa. 2025. Available: https://resourcecentre.savethechildren.net/pdf/SCUS-Kenya-CLIMSEDIS-Report.pdf

[38] OpenStreetMap. OSM History Dump © OpenStreetMap Contributors. [cited 2026 Oct 16]. Available: https://planet.openstreetmap.org/planet/full-history/

[39] Humanitarian Data Exchange | Find & Use Crisis Data | HDX. [cited 19 Oct 2026]. Available: https://data.humdata.org/

[40] Code for Africa. openAFRICA. [cited 2026 Oct 19]. Available: https://open.africa/

