# Supplementary material for: Development of a climate-sensitive disease forecasting tool in the Horn of Africa: A multi-stakeholder exploration of priorities in Ethiopia, Kenya, Somalia, and Uganda
Source: PLOS Glob Public Health. 2026 Nov 10;6(11):e0005634. doi: 10.1371/journal.pgph.0005634 (PMC13750593; doi:10.1371/journal.pgph.0005634)
Supplement: Supplementary file 1 [file pgph.0005634.s001.pdf]

CLIMSEDIS PROJECT

INTERVIEW QUESTION GUIDE

Question guide for ClimSeDis interviews 2024

Climate Sensitive Disease in the Horn of Africa: utility and design of decision support tools

Participant information

1. Name
2. Country of work [geographical location]
3. What is your place of work [organization/programme]?
4. What is your work title [position name]?
5. What activities are you primarily responsible for at work?
6. What populations do you serve? – Urban / rural / other

Diseases of primary concern

7. Are diseases or health conditions in general, the primary focus to your organisation?
   a) If yes, which diseases or health conditions does your organization focus on and which ones do you consider climate-sensitive?
8. What are the three primary climate-sensitive diseases of concern to your organisation?
   [List top three/ can then prompt or list any others stated]
9. What are the challenges faced in preparing for the future risk of these [three] climate-sensitive diseases of primary concern?
   a) Are there any potential solutions to mitigate these challenges?

Decision-making processes

10. At what institutional level are decisions made in relation to climate-sensitive disease surveillance and control? *[Open text - aim to get departmental level information e.g. central/regional/etc.]*
11. Who/which department makes the decisions relating to future disease risks in your institution?
    a) Is the decision process the same for making the decision and communicating the decision on future disease risk?
    b) If no - then who is responsible for communicating these decisions? *[Get the role of person responsible/their level]*

12. How has a specific decision or communication influenced a future disease risk in the national/sub-national? *[Prompt for a specific example]*
    a) Did the decision lead to an outcome that was positive or negative [in general], or you cannot say/don't know? *[Prompt for example]*
13. In terms of timing of regarding a future disease risk – what would be an optimal lead-time to get information of an impending risk or event and deploy intervention strategies. E.g., 1 – 2 – 3 months ahead? *[Prompt to get information in months - not days/weeks as that is not possible]*
14. What is the relationship between national and sub-national (e.g. county/region/district) level decision-making concerning climate-sensitive disease surveillance, control, and response to outbreaks? *[Ensure answer is related to disease risk]*
15. What are the limitations or opportunities to strengthen the connection/relationship between national & subnational level decision-making in relation to climate-sensitive disease surveillance, control, and response to outbreaks.]? *[Ensure answer is related to disease risk]*
16. What is the line (draw diagram if needed) between decisions made on disease intervention/ outbreak response at the higher level and the implementation of the actions on the ground (community/district/county level)? *[May need to have paper ready to draw]*

Climate and Environment

17. Do you use climate or environmental data in relation to diseases risk in any way? *[Prompt e.g. distributions or outbreaks or when to distribute interventions.]*
    a) If yes, are there any specific weather/climate factors that you / your organization/team monitor or use [e.g. rainfall] to guide of potential risk?
    b) If so, where do you get your information? Any connection with environmental / meteorological offices in the country?
    c) If no, what is the reason for not using any specific weather/climate data? *[Prompt e.g. don't know how to use/no expertise/not important]*
18. Have you heard of the El Nino Southern Oscillation – referred to as ENSO [or El Nino or la Nina] sometimes?
    a) If yes – What is your understanding of it and how has it or currently influences the climate and risk of diseases and outbreaks in your region of work?
    [Prompt – El Nino understanding and how it has affected them/their work/ in last few years – aim to get specific example]

b) To your knowledge, are there any diseases that are more affected by the ENSO/La Nina large-scale climate cycle than others in your country/region of work?

Existing data and tools to support decision-making

19. What information sources feed into institutional decision-making about future disease risks? *[Prompt e.g. epidemiological data]*
20. What information about specific diseases do you collect for decision-making processes? Where does the data come from? *e.g. clinics, hospitals, database [Describe - e.g. daily/ community level/case numbers]*
21. Is the climate data integrated with disease data to support making specific decisions at present in your area of work or organization? *[Y/N]*
   a) If yes, what sources of empirical data on climate and disease are integrated into your decision-making?
22. Does seasonality factor into routine decisions in your institutional environment? *[Y/N]*
   a) If yes, what season[s] are important in relation to what diseases/ and do different seasons pose different disease risks.
23. Have you ever used a modelled output related to the risk of disease or a climate/weather event for decision-making? *[Y/N][Prompt-For example, a weather forecast or disease map on a website.]*
   a) If yes, which modelled output? *[Prompt-did it include disease or climate or other factors]*
   b) If no, would you consider them acceptable for use in decision-making on future disease risks [if a tool was made user-friendly]?
24. What reporting systems [paper-based or electronic/digital] are used to assist decisions in climate-sensitive disease risks in deployment of interventions/response to outbreaks?
   a) What are the strengths and weaknesses of the systems being used for disease surveillance and control?
   Write answers separately for
   Paper based tools - Strengths - Weakness
   Digital tools – Strengths – Weakness
25. Can you describe in more detail the electronic/digital tools used or integrated into the decision process and how does the process flow?
   a) Is this/are these digital tool[s] openly accessible to your whole organization and to the general public?
   b) If yes – prompt for a web link

c) Is this/are these digital tools easy to use? Was any training required to use? If yes to training - describe the training

26. If only paper-based reporting system in place – can you explain why there is no electronic/digital system in place? *Prompt for reason e.g. costs/ capacity / Would they use if available? What would be useful to enhance what they have?*

The format and presentation of digital tools

27. Do you/have you ever had a digital tool / web interface to interact with to get better information about future disease risk and /or climate? *[Ask about both disease and climate]*

a) If yes, was it a phone, tablet or computer or a mix?

28. In terms of digital tool - What is or would be the preferred format for analysing, displaying and reporting data for decisions on future disease risks?

[Prompt to describe each one i) analysing ii) displaying iii) reporting]

29. How do you think visual aids (e.g. maps, graphs) are of added value to decision making in relation to future disease risk?

30. *We are developing a new digital tool using climate to help identify in advance – where an increased risk or outbreak of disease may occur at sub-national level – the following questions are related to the interface/dashboard and the information it could contain.*

[Refer to /show screenshots of example dashboard]

a) What would you and/or your team prefer to use in terms of the device– e.g. a phone, tablet, or computer or a mix?

b) What type of disease data would be useful to have in advance?

[Prompt for e.g. potential case numbers/ prevalence/ populations at risk]

c) The tool will display maps - What geographical resolution/ or at subnational level would be useful to interact with to help make decisions and communicate responses/actions?

[Prompt for a higher than village level admin level e.g. health catchment, sub-district, district, county, state]

d) What level of interaction would you like with the tool/ dashboard/- i.e., what things would be useful to do or get out of it to help make decisions and communicate responses/actions regarding future disease risks?

[Describe -prompt zooming/extract data/ graphs for reports]

e) Any other comments regarding tool use/preference/?

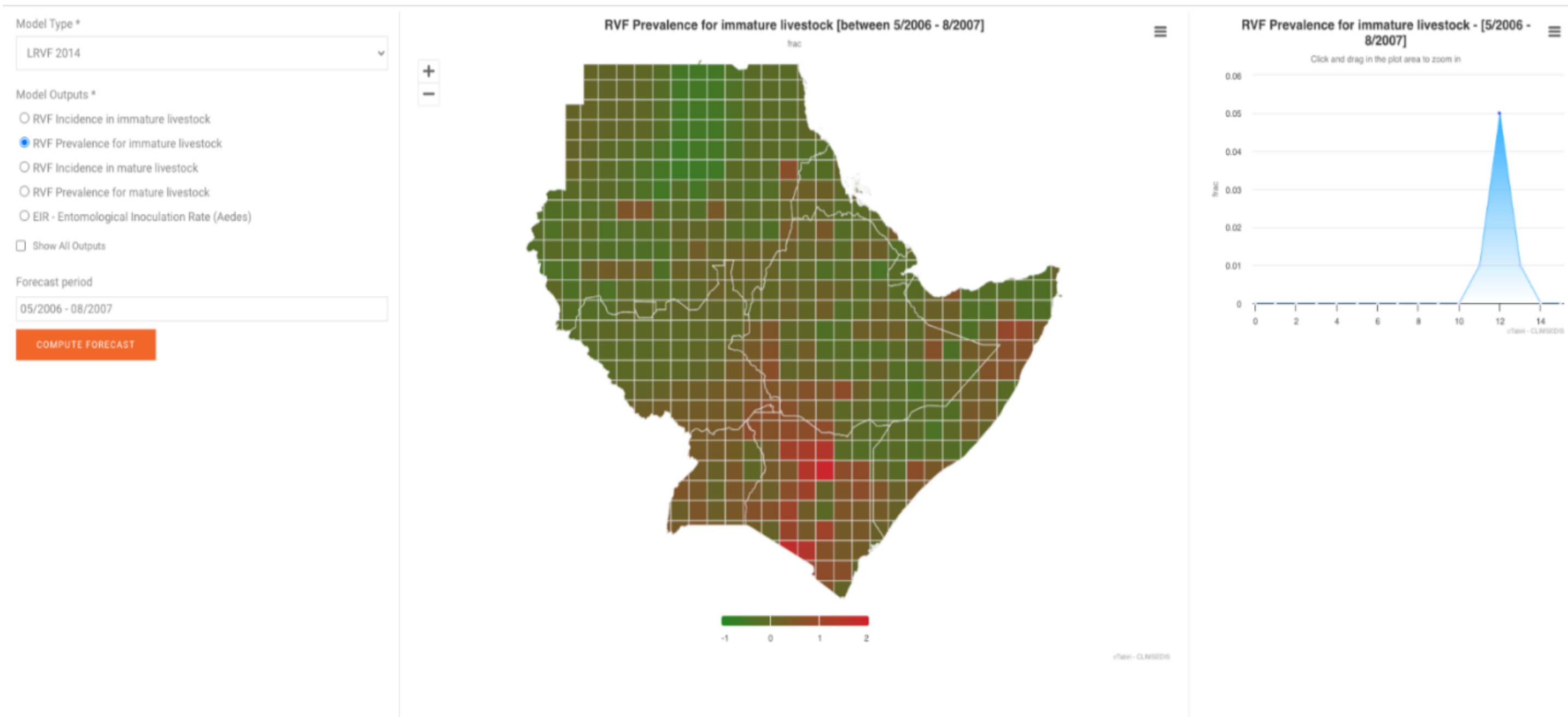

Sustainability

31. What do you think the main barriers are / or would be to sustaining the use and maintenance of a new digital tool that would be available on a mobile phone or computer desktop?

a) What would be possible solutions or ways to address the challenges related to sustainability?

Other

32. Is there anything else you want to highlight in relation to digital tools used for making decisions on climate sensitive diseases/outbreaks/control/surveillance/outbreaks [in your organization]?

CLIMSEDIS Survey tool - Questionnaire template

Sections of the questionnaire

a) Participant information (gender / work role/ level)
b) Decision-making and its consequences
c) Existing tools to support decision-making
d) Diseases of primary concern
e) The format and presentation of an eventual tool
f) Sustainability

No.	Question	Answer
**Section 1 - Participant information**		
1.1	Name	
1.2	Country of work [geographical location]	
1.3	What is your place of work [organization/programme]?	
1.4	What is your work title [position name]?	
1.5	What activities are you primarily responsible for at work?	
1.6	[What populations do you serve? – urban / rural / other]	
**Section 2 - Diseases of primary concern**		
2.1	Are diseases or health conditions in general, the primary focus to your organization?<br>*[Y/N]*	
2.1.1	If yes, which diseases or health conditions does your organization focus on and which ones do you consider climate sensitive?	
2.2	What are the three primary climate-sensitive diseases of concern to your organization?<br>*[List top three/ can then prompt or list any others stated]*	
2.3	What are the challenges faced in preparing for the future risk of these [three] climate-sensitive diseases of primary concern?	
2.3.1	Are there any potential solutions to mitigate these challenges?	

2.4	What other challenges do you encounter?	
**Section 3 – Decision-making processes**		
3.1	At what institutional level are decisions made in relation to climate-sensitive disease surveillance and control?<br>*[Open text - aim to get departmental level information e.g. central/regional/etc.]*	
3.2	Who/which department makes the decisions relating to future disease risks in your institution?	
3.2.1	Is the decision process the same for making the decision and communicating the decision on future disease risk?<br>*[Y/N]*	
3.2.2	If no - then who is responsible for communicating these decisions?<br>*[Get the role of person responsible/their level]*	
3.3	How has a specific decision or communication influenced a future disease risk in the national/sub-national?<br>*[Prompt for a specific example]*	
3.3.1	Did the decision lead to an outcome that was positive or negative [in general]? Or you cannot say/don't know?<br>*[Prompt for example]*	
3.4.	In terms of timing of regarding a future disease risk – what would be an optimal lead-time to get information of an impending risk or event to help deploy intervention strategies? e.g., 1 - 2 - 3- 6 months ahead.<br>*[Prompt to get information in <u>months</u> - not days/weeks as that is not possible]*	
3.5	What is the relationship between national and sub-national (e.g. county/region/district) level decision-making concerning climate-sensitive disease surveillance, control, and response to outbreaks?<br>*[Ensure answer is related to disease risk]*	
3.6	What are opportunities to strengthen the connection between national & subnational level decision-making [in relation to climate-sensitive disease surveillance, control, and response to outbreaks]?	

	[Ensure answer is related to disease risk]	
3.7	What is the line (draw diagram if needed) between decisions made on disease intervention/ outbreak response at the higher level and the implementation of the actions on the ground (community/district/county level)?<br>*[May need to have paper ready to draw]*	
**Section 4 – Climate and Environment**		
4.1	Do you use climate or environmental data in relation to diseases risk in any way?<br>*[Prompt e.g. distributions or outbreaks or when to distribute interventions]*	
4.1.1	If yes, are there any specific weather/climate factors that you / your organization/team monitor or use [e.g. rainfall] to guide of potential risk?	
4.1.2	If so, where do you get your information? Any connection with environmental / meteorological offices in the country?	
4.1.3	If no, what is the reason for not using any specific weather/climate data?<br>*[Prompt e.g. don't know how to use/no expertise/not important]*	
4.2	Have you heard of the El Nino Southern Oscillation – referred to as ENSO [or El Nino or la Nina] sometimes?<br>*[Y/N]*	
4.2.1	If yes – What is your understanding of it and how has it or currently influences the climate and risk of diseases and outbreaks in your region of work?<br>*[Prompt – El Nino understanding and how it has affected them/their work/ in last few years – aim to get specific example]*	
4.2.2	<u>To your knowledge</u>, are there any diseases that are more affected by the ENSO/La Nina large-scale climate cycle than others in your country/region of work?	
**Section 5 - Existing data and tools to support decision-making**		
5.1	What information sources feed into institutional decision-making about future climate-sensitive disease risk?<br>*[Prompt e.g. epidemiological data]*	

5.2	What information about specific diseases do you collect for decision-making processes? Where does the data come from? e*.g. clinics, hospitals, database*<br>*[Describe - e.g. daily/ community level/case numbers]*	
5.3	Is the climate data integrated with disease data to support making specific decisions at present in your area of work or organization?<br>*[Y/N]*	
5.3.1	If yes, what sources of empirical data on climate and disease are integrated into your decision-making?	
5.4	Does seasonality factor into routine decisions in your institutional environment?<br>*[Y/N]*	
5.4.1	If yes, what season[s] are important in relation to what diseases/ and do different seasons pose different disease risks.	
5.5	Have you ever used a modelled output related to the risk of disease or a climate/weather event for decision-making?<br>*[Y/N][Prompt-For example, a weather forecast or disease map on a website.]*	
5.5.1	If yes, which modelled output?<br>*[Prompt-did it include disease or climate or other factors]*	
5.5.2	If no, would you consider them acceptable for use in decision-making on future disease risks [if a tool was made user-friendly]?	
5.6	What reporting systems [paper-based or electronic/digital] are used to assist decisions in climate-sensitive disease risks in deployment of interventions/response to outbreaks?	
5.6.1	What are the strengths and weaknesses of the systems being used for disease surveillance and control?<br>*Write answers separately for*<br>*Paper based tools - Strengths - Weakness*<br>*Digital tools – Strengths – Weakness*	
5.7	Can you describe in more detail the electronic/digital tools used or integrated into the decision process and how does the process flow?	

5.7.1	Is this/are these digital tool[s] openly accessible to your whole organization and to the general public?<br>If yes – prompt for a web link	
5.7.2	Is this/are these digital tools easy to use? Was any training required to use? If yes to training - describe the training	
5.8	If only paper-based reporting system in place – can you explain why there is no electronic/digital system in place?<br>*Prompt for reason e.g. costs/ capacity / Would they use if available? What would be useful to enhance what they have?*	
**Section 6 - The format and presentation of digital tools**		
6.1	Do you/have you ever had a digital tool / web interface to interact with to get better information about future disease risk and /or climate?<br>*[ask about both disease and climate]*	
6.1.1	If yes, was it a phone, tablet or computer or a mix?	
6.2	In terms of digital tool - What is or would be the preferred format for analyzing, displaying and reporting data for decisions on future disease risks?<br>*[Prompt to describe each one i) analyzing ii) displaying iii) reporting]*	
6.3	How do you think visual aids (e.g. maps, graphs) are of added value to decision making in relation to future disease risk?	
6.4	*We are developing a new digital tool using climate to help identify in advance – where an increased risk or outbreak of disease may occur at sub-national level – the following questions are related to the interface/dashboard and the information it could contain.*<br>*[Refer to /show screenshots of example dashboard]*	
6.4.1	What would you and/or your team prefer to use in terms of the device– e.g. a phone, tablet, or computer or a mix?	
6.4.2	What type of disease data would be useful to have in advance?<br>*[Prompt for e.g. potential case numbers/ prevalence/ populations at risk]*	

6.4.3	The tool will display maps - What geographical resolution/ or at subnational level would be useful to interact with to help make decisions and communicate responses/actions?<br>[*Prompt for a higher than village level admin level e.g. health catchment, sub-district, district, county, state*]	
6.4.4	What level of interaction would you like with the tool/ dashboard/- i.e., what things would be useful to do or get out of it to help make decisions and communicate responses/actions regarding future disease risks?<br>[*Describe -prompt zooming/extract data/ graphs for reports]*	
6.4.5	Any other comments regarding tool use/preference/?	
	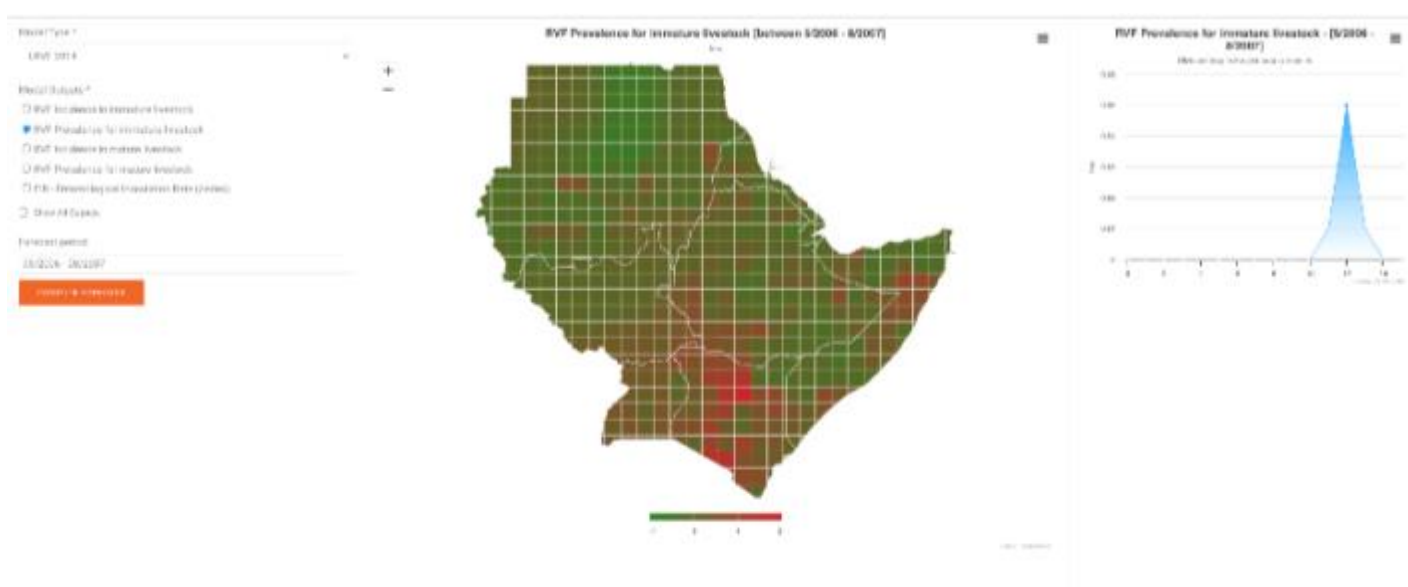	
	**Section 7 - Sustainability**	
7.1	What do you think the main barriers are / or would be to sustaining the use and maintenance of a new digital tool that would be available on a mobile phone or computer desktop?	
7.1. 1	What would be possible solutions or ways to address the challenges related to sustainability?	
	**Other**	
	Is there anything else you want to highlight in relation to digital tools used for making decisions on climate sensitive diseases/outbreaks/control/surveillance/outbreaks [in your organization]?	

Thank you for your time
