# Supplementary material for: Development of a climate-sensitive disease forecasting tool in the Horn of Africa: A multi-stakeholder exploration of priorities in Ethiopia, Kenya, Somalia, and Uganda
Source: PLOS Glob Public Health. 2026 Nov 10;6(11):e0005634. doi: 10.1371/journal.pgph.0005634 (PMC13750593; doi:10.1371/journal.pgph.0005634)
Supplement: Supplementary file 3 [file pgph.0005634.s002.docx]

# **S1 Appendix.**

**Table A.** Countries and organisational affiliations of stakeholders participating in Key Informant Interviews (*N*=63).

|  | **Country** | | | | **Total, *N*=63** |
| --- | --- | --- | --- | --- | --- |
|  | **Ethiopia, *N*=12** | **Kenya, *N*=21** | **Somalia, *N*=14** | **Uganda, *N*=16** |  |
| **Stakeholder group, *n* (%)** | | | | | |
| **Government** | 7 (58.3) | 8 (38.1) | 5 (35.7) | 9 (56.3) | 29 (46.0) |
| Health or Public Health | 7 (58.3) | 2 (9.5) ^a^ | 1 (7.1) | 4 (25.0) ^b^ | 14 (22.2) |
| Veterinary or Agriculture | - | 3 (14.3) ^c^ | 2 (14.3) | 3 (18.8) | 8 (12.7) |
| Environment, Climate or Water | - | 1 (4.8) | 1 (7.1) | 1 (6.3) | 3 (4.8) |
| Meteorology | - | 1 (4.8) | - | 1 (6.3) | 2 (3.2) |
| Zoonotic Diseases | - | 1 (4.8) | - | - | 1 (1.6) |
| Disaster Preparedness and Food | - | - | 1 (7.1) | - | 1 (1.6) |
| **NGO/ Non-profit organisation** | 1 (8.3) | 4 (19.0) | 3 (21.4) | 3 (18.8) | 11 (17.5) |
| **Multi-lateral agency: UN** | 1 (8.3) | 3 (14.3) | 2 (14.3) | 2 (12.5) | 8 (12.7) |
| **Academia** | 1 (8.3) | 2 (9.5) | 3 (21.4) | 1 (6.3) | 7 (11.1) |
| **International research organisation** | - | 3 (14.3) | - | 1 (6.3) | 4 (6.3) |
| **Multi-lateral agency: Non-UN** | 1 (8.3) | - | - | - | 1 (1.6) |
| **Private sector** | - | - | 1 (7.1) | - | 1 (1.6) |
| **Multiple affiliations** | 1 (8.3) ^d^ | 1 (4.8) ^e^ | - | - | 2 (3.2) |

Acronyms: NGO=Non-Governmental Organisation

^a^ Including virus research institute (*n*=1); ^b^ Including virus research institute (*n*=1); ^c^ Including veterinary research institute (*n*=1); ^d^ UN agency and government (Planning and Development; *n*=1); ^e^ UN agency, other multi-lateral agency, and academia (*n*=1).

**Table B.** Countries and organisational affiliations of stakeholders participating in Focus Group Discussions (*N*=57).

|  | **Country** | | | **Total, *N*=57** |
| --- | --- | --- | --- | --- |
|  | **Ethiopia, *N*=13** | **Somalia, *N*=25** | **Uganda, *N*=19** |  |
| **Stakeholder group, *n* (%)** | | | | |
| **Government** | 1 (7.7) | 13 (52.0) | 18 (94.7) | 32 (56.1) |
| Health or Public Health | 1 (7.7) | 13 (52.0) | 5 (26.3) | 19 (33.3) |
| Veterinary or Agriculture | - | - | 4 (21.1) | 4 (7.0) |
| Meteorology | - | - | 1 (5.3) | 1 (1.8) |
| Environment | - | - | 2 (10.5) | 2 (3.5) |
| One Health | - | - | 2 (10.5) | 2 (3.5) |
| Entomology | - | - | 1 (5.3) | 1 (1.8) |
| Community Development | - | - | 1 (5.3) | 1 (1.8) |
| District-level administration | - | - | 1 (5.3) | 1 (1.8) |
| Laboratory | - | - | 1 (5.3) | 1 (1.8) |
| **NGO** | - | 9 (36.0) | - | 9 (15.8) |
| **Academia ^a^** | 12 (92.3) | 3 (12.0) | 1 (5.3) | 16 (28.1) |

Acronyms: NGO=non-Governmental Organisation

^a^ Assistant Professor, lecturer, and researcher positions.

**Table C.** Challenges in climate-sensitive disease preparedness reported by Key Informant Interview participants (*N*=63).

| **Challenges in disease preparedness** | **Stakeholder reports, *n* (%), *N*=63** |
| --- | --- |
| Financial resources | 26 (41.3) |
| Data availability, access, quality, or sharing | 21 (33.3) |
| Information sharing, collaboration, or coordination between sectors or stakeholders | 11 (17.5) |
| Material resources (including laboratory diagnostics and vaccines) | 12 (19.0) |
| Human resources or capacity | 11 (17.5) |
| Risk model or prediction tool availability, accuracy, use, or knowledge | 10 (15.9) |
| Disease surveillance capacity (including passive surveillance systems) | 9 (14.3) |
| Disease forecasting or prediction | 6 (9.5) |
| Health infrastructure | 6 (9.5) |
| Variable knowledge, capacity, or communication between sectors or stakeholders | 6 (9.5) |
| Resources (not specified) | 5 (7.9) |
| Community-level knowledge or risk perceptions related to climate-sensitive diseases | 5 (7.9) |
| Increase in emerging diseases | 3 (4.8) |
| Environment or weather event surveillance | 3 (4.8) |
| Recognition of links between weather or climate events and disease | 3 (4.8) |
| Climate change or increase in climate variability | 3 (4.8) |
| Human population increase or movement | 2 (3.2) |
| Access to disease recommendations | 2 (3.2) |
| Lack of national disease plans | 2 (3.2) |
| Sub-national or rural area capacity | 2 (3.2) |
| Climate or disease leadership or organisational infrastructure | 2 (3.2) |
| Failure of control or prevention measures | 2 (3.2) |
| Lack of community engagement or ‘buy-in’ | 2 (3.2) |
| Political instability or conflict | 2 (3.2) |
| Other ^a^ | 8 (12.9) |

^a^ For the following ‘other’ challenges, *n*=1: Accuracy of notifications; sustainability; trade sensitive nature of disease; vector surveillance; variable preparedness for different diseases; research capacity; not primary focus of organisation; logistical constraints (including access to rural areas).

**Table D.** Diseases more affected by the El Niño-Southern Oscillation (ENSO) or La Niña events, as reported by Key Informant Interview participants (*N*=63).

|  | **Country** | | | | **Total, *N*=63** |
| --- | --- | --- | --- | --- | --- |
|  | **Ethiopia, *N*=12** | **Kenya, *N*=21** | **Somalia, *N*=14** | **Uganda, *N*=16** |  |
| **Stakeholder reports, *n* (%)** | | | | | |
| Malaria | 1 (8.3) | 7 (33.3) | 7 (50.0) | 9 (56.3) | 24 (38.1) |
| Cholera or diarrhoeal disease | 1 (8.3) | 7 (33.3) | 6 (42.9) | 6 (37.5) | 20 (31.7) |
| RVF | - | 9 (42.9) | - | 3 (18.8) | 12 (19.0) |
| Mosquito or vector borne disease ^a^ | - | 7 (33.3) | 4 (28.6) | - | 11 (17.5) |
| Anthrax | - | 1 (4.8) | - | 8 (50.0) | 9 (14.3) |
| Dengue fever | 1 (8.3) | 3 (14.3) | 3 (21.4) |  | 7 (11.1) |
| Respiratory diseases | - | 3 (14.3) | 3 (21.4) | 1 (6.3) | 7 (11.1) |
| Typhoid fever | - | - | 1 (7.1) | 3 (18.8) | 4 (6.3) |
| Waterborne diseases ^a^ | - | - | 4 () | - | 4 (6.3) |
| CCHF | - | - | - | 3 (18.8) | 3 (4.8) |
| Chikungunya | - | 1 (4.8) | 1 (7.1) | 1 (6.3) | 3 (4.8) |
| Zika | - | 2 (9.5) | - | - | 2 (3.2) |
| Other | - | 5 (23.8) ^b^ | 1 (7.1) ^c^ | 5 (31.3) ^d^ | 11 (17.5) |

Acronyms: CCHF=Crimean Congo Haemorrhagic Fever; RVF=Rift Valley Fever.

^a^ Diseases within these categories not specified.

For the following diseases, *n*=1: ^b^ Dysentery; bluetongue; trypanosomiasis; lumpy skin disease; leishmaniasis; ^c^ Yellow fever; ^d^ Influenza; Marburg; West Nile virus; schistosomiasis; yellow fever.

**Table E.** Institutional levels of decision-making in relation to climate-sensitive disease surveillance and control, reported by Key Informant Interview participants (*N*=63).

| **Institutional level** | **Stakeholder reports, *n* (%), *N*=63** |
| --- | --- |
| Central: Ministry or institution ^a^ | 24 (38.1) |
| Central: Department or directorate | 11 (17.5) |
| Sub-national ^b^ | 6 (9.5) |
| Program or project ^c^ | 4 (6.3) |
| International level ^d^ | 1 (1.6) |
| Regional (multi-country) level ^d^ | 1 (1.6) |

^a^ Includes stakeholder responses: Central, Ministry, “*institution head*”, and “*top level*”.

^b^ Includes stakeholder responses: Sub-national, district, regional (referring to sub-national administrative unit).

^c^ Includes stakeholder responses: Program, project dependent, and donor dependent.

^d^ NGO (*n*=1) with international and regional (multi-country) offices.

**Table F.** Relationships between national and sub-national level decision-making on climate-sensitive diseases, as reported by Key Informant Interview and Focus Group Discussion participants.

| **Country** | **Relationship characteristics** | **Challenges** | **Opportunities or solutions** |
| --- | --- | --- | --- |
| **Ethiopia** | Decision-making is decentralised and involves multiple stakeholders. Key decisions made at federal (national) level, with implementation at sub-national (district, zonal, and regional) levels. | - Coordination between stakeholders fragmented, leading to delays in response. - Delays at different levels of flow of information. - Decision-making capacity varies between regions (related to variable technical expertise). - Frontline workers lack authority to initiate rapid responses. | - Availability of advanced tools for informed decision-making. - Partnerships with key organisations. - Stronger collaboration between stakeholders to improve coordination. - Establishment of climate-resilient facilities to provide technical and logistical support for disease risk management. - Financial support for training and sub-national level support and monitoring. |
| **Kenya** | Decision-making is influenced by the devolved system of governance between the national and county government. National government is mostly involved in policy formulation and regulation while county government is involved in implementation. Some decisions are made at the national level while some remain devolved. | - Communication challenges. - Chain of command unclear. - Misalignment in coordination of activities. - Better capacity at national level to implement strategies. | - Capacity building (including entomology). - Promotion of harmonised working conditions. - Address localised challenges, raising awareness of county climate change committees and County One Health Units. - Improved communication to allow information flow. |
| **Somalia** | Decision-making is centralised at national level. Directives flow from key sectors through regional and district offices to frontline health workers. District authorities feedback to national level to inform and refine decisions. | - Gaps in effective implementation and monitoring on the ground. - Community needs not effectively incorporated into national planning. - District offices lack resources to execute policies. - Ineffective coordination and communication between national and district offices. | - Strengthen governance structures (e.g., define institutional mandates, establish an integrated One Health response). - Formal communication channels and joint planning activities between national and district levels. - District capacity building to build technical expertise. - Decentralised resource allocation. - Strengthen data systems. - Strengthen community engagement to address community needs. |
| **Uganda** | Decision-making is centralised at national level. Sub-national levels focus on policy implementation and enforcement but are involved in national task forces during outbreak responses. | - Gaps in implementation of national decisions at sub-national levels, related to resource limitations and lack of support systems for field implementation. - Poor monitoring and evaluation of activities. | - Cross-sectoral collaboration. - Multidisciplinary collaborative platforms (e.g., National One Health platform). - Inclusive task forces (e.g., inclusion of district representatives on national task forces). - Availability of trained personnel at all organisational levels. |

**Table G.** Sources of data collected for institutional decision-making about future disease risks, reported by Key Informant Interview participants (*N*=63).

| **Data sources** | **Stakeholder reports, *n* (%), *N*=63** |
| --- | --- |
| Hospital/ health facility | 23 (36.5) |
| Community/ CBO | 13 (20.6) |
| Government or official country data | 11 (17.4) |
| Surveillance personnel or system | 7 (11.1) |
| Satellite or meteorology source | 4 (6.3) |
| Environment or weather stations | 2 (3.2) |
| International organisation or partner | 2 (3.2) |
| Media (newspaper or social media) | 2 (3.2) |
| Research or expert opinion | 2 (3.2) |
| NGO | 1 (1.6) |

Acronyms: CBO=community-based organisation; NGO=non-governmental organisation.

**Table H.** Strengths and weaknesses of electronic and paper-based systems for disease surveillance and control, reported by Key Informant Interview participants (*N*=63).

| **System** | **Strengths ^a^** | **Weaknesses ^a^** |
| --- | --- | --- |
| **Electronic** | Faster or real-time reporting for rapid decision-making | Requires mobile device or Internet/ electricity access |
|  | Wider dissemination or easier data sharing | Lack of data uniformity or data cleaning requirements |
|  | Improved accessibility (e.g., via mobile device) | Resource requirements (material or financial) |
|  | Improved data accuracy and quality | Technological skill or training requirements |
|  | Improved data security | Data security issues |
|  | More reliable | System maintenance |
|  | Increased capacity for data analysis | Data management requirements |
|  | Reduced physical storage requirements |  |
|  | Easier to enter data |  |
|  | Improved data visualisations (e.g., maps) |  |
| **Paper-based** | Easy to understand and use | Takes time |
|  | Low cost (including maintenance costs) | No back-up files or copies |
|  | No reliance on electricity or Internet access | Bulky or physical storage issues |
|  | No risk of hacking or system corruption | Environmental impact of paper use |
|  | Easy to customise | Data validation issues or human error |
|  | Easy access | Limited data analysis |
|  |  | Human resource requirements |

^a^ Reported by ≥2 participants.
